# How the Post-Data Severity Converts Testing Results into Evidence for or against Pertinent Inferential Claims

**DOI:** 10.3390/e26010095

**Published:** 2024-01-22

**Authors:** Aris Spanos

**Affiliations:** Department of Economics, Virginia Tech, Blacksburg, VA 24061, USA; aris@vt.edu

**Keywords:** replication, untrustworthy evidence, statistical misspecification, statistical vs. substantive significance, pre-data vs. post-data error probabilities, p-hacking, post-data severity evaluation, observed confidence intervals, effect sizes

## Abstract

The paper makes a case that the current discussions on replicability and the abuse of significance testing have overlooked a more general contributor to the untrustworthiness of published empirical evidence, which is the uninformed and recipe-like implementation of statistical modeling and inference. It is argued that this contributes to the untrustworthiness problem in several different ways, including [a] statistical misspecification, [b] unwarranted evidential interpretations of frequentist inference results, and [c] questionable modeling strategies that rely on curve-fitting. What is more, the alternative proposals to replace or modify frequentist testing, including [i] replacing *p*-values with observed confidence intervals and effects sizes, and [ii] redefining statistical significance, will not address the untrustworthiness of evidence problem since they are equally vulnerable to [a]–[c]. The paper calls for distinguishing between unduly data-dependant ‘statistical results’, such as a point estimate, a *p*-value, and accept/reject $H_{0}$ , from ‘evidence for or against inferential claims’. The post-data severity (SEV) evaluation of the accept/reject $H_{0}$ results, converts them into evidence for or against germane inferential claims. These claims can be used to address/elucidate several foundational issues, including (i) statistical vs. substantive significance, (ii) the large n problem, and (iii) the replicability of evidence. Also, the SEV perspective sheds light on the impertinence of the proposed alternatives [i]–[iii], and oppugns [iii] the alleged arbitrariness of framing $H_{0}$ and $H_{1}$ which is often exploited to undermine the credibility of frequentist testing.

## 1. Introduction

The replication crisis has dominated discussions on empirical evidence and their trustworthiness in scientific journals for the last two decades. The broad agreement is that the non-replicability of such empirical evidence provides prima facie evidence of their untrustworthiness; see National Academy of Sciences [1], Wasserstein and Lazar [2], Baker [3], Hoffler [4]. A statistical study is said to be replicable if its empirical results can be independently confirmed – with very similar or consistent results – by other researchers using akin data and modeling the same phenomenon of interest.

Using the Medical Diagnostic Screening (MDS) perspective on Neyman-Pearson (N-P) testing Ioannidis [5] declares “… most published research findings are false”, attributing the untrustworthiness of evidence to several abuses of frequentist testing, such as p-hacking, multiple testing, cherry-picking and low power. This diagnosis is anchored on apparent analogies between the type I/II error probabilities and the false negative/positive probabilities of the MDS model, tracing the untrustworthiness to ignoring the Bonferroni-type adjustments needed to ensure that the *actual* error probabilities approximate the *nominal* ones. In light of that, leading statisticians in different applied fields called for reforms which include replacing *p*-values with observed Confidence Intervals (CIs), using effect sizes, and redefining statistical significance; see Benjamin et al. [6].

In the discussion that follows, a case is made that Ioannidis’ assessment about the untrustworthiness of published empirical findings is largely right. Still, the veracity of viewing the MDS model as a surrogate for N-P testing, and its pertinence in diagnosing the untrustworthiness of evidence problem are highly questionable. This stems from the fact that the invoked analogies between the type I/II error probabilities and the false negative/positive probabilities of the MDS model are more apparent than real, since the former are hypothetical/unobservable/unconditional and the latter are observable conditional probabilities; see Spanos [7].

A more persuasive case can be made that the untrustworthiness of empirical evidence stems from the broader problem of *the uninformed and recipe-like implementation of statistical modeling and inference* that contributes to untrustworthy evidence in several interrelated ways, including:

[a] Statistical misspecification: invalid probabilistic assumptions imposed (implicitly or explicitly) on one’s data, comprising the invoked statistical model $M_{\theta }\left(x\right)$ .

[b] ‘Empirical evidence’ is often conflated with raw ‘inference results’, such as point estimates, effect sizes, observed CIs, accept/reject $H_{0}$ results, and *p*-values, giving rise to (i) erroneous evidential interpretations of these results, and (ii) unwarranted claims relating to their replicability.

[c] Questionable modeling strategies that rely on curve-fitting of hybrid models—an amalgam of substantive subject matter and probabilistic assumptions—guided by error term assumptions and evaluated on goodness-of-fit/prediction grounds. The key weakness of this strategy is that excellent goodness-of-fit/prediction is neither necessary nor sufficient for the statistical adequacy of the selected model since it depends crucially on the invoked loss function whose choice is based on information other than the data. It can shown that statistical models chosen on goodness-of-fit/prediction grounds are often statistically misspecified; see Spanos [8].

Viewed in the broader context of [a]–[c], the abuses of frequentist testing represent the tip of the untrustworthy evidence iceberg. It also questions the presumption that replicability attests to the trustworthiness of empirical evidence. As argued by Leek and Peng [9]: “… an analysis can be fully reproducible and still be wron.” (p. 1314). For instance, dozens of MBA students confirm the efficient market hypothesis (EMH) on a daily basis because they follow the same uninformed and recipe-like, implementation of statistics, unmindful of what it takes to ensure the trustworthiness of the ensuing evidence by addressing the issues [a]–[c]; see Spanos [10].

The *primary focus* of the discussion that follows is on [b] with brief comments on [a] and [c], but citing relevant published papers. The discussion revolves around the distinction between unduly data-specific ‘inference results’, such as point estimates, observed CIs, *p*-values, effect sizes, and the accept/reject $H_{0}$ results, and ensuing inductive generalizations from such results in the form of ‘evidence for or against germane inferential claims’ framed in terms of the unknown parameters θ. The crucial difference between ‘results’ and ‘evidence’ is twofold:

(a) the evidence is framed in terms of *post-data* error probabilities aiming to account for the uncertainty arising from the fact that ‘inference results’ rely unduly on the particular data $x_{0}:=\left(x_{1},x_{2},\ldots ,x_{n}\right),$ which constitutes a single realization $X\;\;=\;\;x_{0}$ of the sample $X:=(X_{1},\ldots ,X_{n})$ , and

(b) the evidence, in the form of warranted inferential claims, enhances learning from data $x_{0}$ about the stochastic mechanism that could have given rise to this data.

As a prelude to the discussion that follows, Section 2 provides a brief overview of Fisher’s model-based frequentist statistics with special emphasis on key concepts and pertinent interpretations of inference procedures that are invariably misconstrued by the uninformed and recipe-like implementation of statistical modeling and inference. Section 3 discusses a way to bridge the gap between unduly data-specific inference results and an evidential interpretation of such results, in the form of the post-data severity (SEV) evaluation of the accept/reject $H_{0}$ results. The SEV evaluation is used to elucidate or/and address several foundational issues that have bedeviled frequentist testing since the 1930s, including the large *n* problem, statistical vs. substantive significance and the replicability of evidence, as opposed to the replicability of statistical results. In Section 4 the SEV evaluation is used to appraise several proposed alternatives to (or modifications of) N-P testing by the replication literature, including replacing the *p*-value with effect sizes and observed CIs and redefining statistical significance. Section 5 compares and contrasts the evidential account based on the SEV evaluation with Royall’s [11] Likelihood Ratio approach to statistical evidence.

## 2. Model-Based Frequentist Inference

### 2.1. Fisher’s Statistical Induction

Fisher [12] pioneered modern frequentist statistics by viewing data $x_{0}$ as a typical realization of a prespecified parametric statistical model whose generic form is:

(1) $$\begin{array}{c} M_{\theta }\left(x\right)\;=\;\left\{f\left(x;\theta \right),\;\theta \in \Theta \subset R^{m},\;x\in R_{X}^{n}\right\},\;m<n,\; \end{array}$$

where Θ and $R_{X}^{n}$ denote the parameter and sample space, respectively, $f\left(x;\theta \right),\;x\in R_{X}^{n},$ refers to the (joint) *distribution of the sample* X . The initial choice (specification) of $M_{\theta }\left(x\right)$ should be a response to the question: “Of what population is this a random sample?” (Fisher, [12], p. 313), underscoring that: ‘the adequacy of our choice may be tested a posteriori’ (ibid., p. 314). This can be secured by establishing the statistical adequacy (approximate validity) of $M_{\theta }\left(x\right)$ using thorough Mis-Specification (M-S) testing; see Spanos [13]. 

**Selecting** $M_{\theta }\left(x\right)$ for data $x_{0}$ has a twofold objective (Spanos [14]):

(i) $M_{\theta }\left(x\right)$ is selected with a view to account for the chance regularity patterns exhibit by data $x_{0}$ by accounting for these regularities using appropriate probabilistic assumptions relating to $\{X_{k},\;t$ **∈** N:=(1,2,…,n,…} from three broad categories: **Distribution** (D), **Dependence** (M) and **Heterogeneity** (H).

(ii) $M_{\theta }\left(x\right)$ is parametrized [ θ ∈ Θ ] in a way that can shed light on the substantive questions of interest using data $x_{0}$ . When such questions are framed in terms of a *substantive model*, say $M_{\varphi }\left(x\right),$ φ ∈ Φ , one needs to bring out the implicit statistical model without restricting its parameters θ, and ensure that θ and φ are related via a set of restrictions g(φ,θ)=0 connecting φ to the data $x_{0}$ via θ .

**Example 1**. Consider the well-known *simple Normal model*:

(2) $$\begin{array}{cc} M_{\theta }\left(x\right): & X_{t}∽\mathrm{NIID}\left(\mu ,\sigma^{2}\right),\;\mu \in R,\;\sigma^{2}\;>\;0,\;x_{t}\in R,\;t\in N:=\left\{1,2,\ldots ,n,\ldots \right\},\; \end{array}$$

where ‘ $X_{t}∽\mathrm{NIID}$ ’ stands for ‘ $X_{t}$ is Normal (D), Independent (M) and Identically Distributed (H)’, R:=(−∞,∞). It is important to emphasize that $M_{\theta }\left(x\right)$ revolves around $f\left(x;\theta \right),\;x\in R_{X}^{n}$ in (1) since it encapsulates all its probabilistic assumptions:

(3) $$\begin{array}{c} f_{SN}\left(x;\theta \right)\overset{I}{=}\prod_{k\;=\;1}^{n}f_{k}\left(x_{k};\theta_{k}\right)\overset{\mathrm{IID}}{\;=\;}\prod_{k\;=\;1}^{n}f\left(x_{k};\theta \right)\overset{\mathrm{NIID}}{\;=\;}{\left(\frac{1}{\sqrt{2\pi \sigma^{2}}}\right)}^{n}\exp\left\{-\frac{1}{2\sigma^{2}}\sum_{k\;=\;1}^{n}{\left(x_{k}-\mu \right)}^{2}\right\},\;x\in R^{n},\; \end{array}$$

and provides the *cornerstone* for all forms of statistical inference.

The *primary objective* of model-based frequentist inference is to ‘learn from data $x_{0}$ ’ about $\theta^{*},$ where $\theta^{*}$ denotes the ‘true’ θ in Θ . This is shorthand for saying that there exists a $\theta^{*}$ ∈ Θ such that $M_{\theta^{*}}\left(x\right)\;=\;f\left({x;\theta }^{*}\right),\;x$ **∈** $R_{X}^{n},$ could have generated $x_{0}$ .

The main variants of statistical inference in frequentist statistics are: (i) point estimation, (ii) interval estimation, (iii) hypothesis testing, and (iv) prediction. These forms of statistical inference share the following features:

(a) They assume that the prespecified statistical model $M_{\theta }\left(x\right)$ is valid vis-à-vis data $x_{0}.$

(b) They aim is to learn about $M_{\theta^{*}}\left(x\right)$ using statistical approximations relating to $\theta^{*}.$

(c) Their inferences are based on a *statistic* (estimator, test statistic, predictor), say $Y_{n}\;=\;g\left(X\right)$ , whose sampling distribution, $f\left(y_{n};\theta \right),\;\forall y\in R_{Y}$ , (∀ stands ‘for all’) is derived directly from the distribution of the sample $f\left(x;\theta \right),\;x\in R_{X}^{n},$ of $M_{\theta }\left(x\right),$ using two different forms of reasoning with prespecified values of θ :

(a) *factual* (estimation and prediction): presume that ${\theta \;=\;\theta }^{*}$ , and

(b) *hypothetical* (testing): $H_{0}$ : $\theta \in \Theta_{0}$ (what if $\theta \in \Theta_{0})$ vs. $H_{1}$ : $\theta \in \Theta_{1}$ (what if $\theta \in \Theta_{1})$ . The crucial difference between these two forms of reasoning is that factual reasoning does not extend to post-data (after $x_{0}$ is known) evaluations relating to evidence, but hypothetical reasoning does. This plays a key role in the following discussion.

The primary role of the sampling distribution of a statistic $f\left(y_{n};\theta \right),\;\forall y\in R_{Y}$ , is to frame the uncertainty relating to the fact that $x_{0}$ is just one, out of all $x\in R_{X}^{n}$ realizations, of X so as to provide (i) the basis for the optimality of the statistic $Y_{n}\;=\;g\left(X\right),$ as well as (ii) the relevant error probabilities to ‘calibrate’ the capacity (optimality) of inference based on $Y_{n}$ ; how often the inference procedure errs.

The *statistical adequacy* (approximate validity) of $M_{\theta }\left(x\right)$ plays a pivotal role in securing the reliability of inference and the trustworthiness of ensuing evidence because it ensures that the *nominal* optimality—derived by assuming the validity of $M_{\theta }\left(x\right)$ —is also *actual* for data $x_{0},$ and secures the approximate equality between the actual (based on $x_{0})$ and the nominal error probabilities. In contrast, when $M_{\theta }\left(x\right)$ is *statistically misspecified*:

(a) the joint distribution of the sample $f\left(x;\theta \right),\;x\in R_{X}^{n},$ and the likelihood function $L\left(\theta ;x_{0}\right)\propto f\left(x_{0};\theta \right),\;\theta \in \Theta ,$ are both erroneous,

(b) all sampling distributions $f(y_{n};\theta ),$ derived by invoking the validity of $f\left(x;\theta \right),\;x\in R_{X}^{n},$ will be incorrect, (i) giving rise to ‘non-optimal’ estimators, and (ii) sizeable *discrepancies* between the actual and nominal error probabilities.

Applying a α=0.05 significance level test when the actual type I error probability is 0.97 due to invalid probabilistic assumptions will yield untrustworthy evidence. Increasing the sample size will often worsen the untrustworthiness by increasing the discrepancy between actual and nominal error probabilities; see Spanos [15], p. 691. Hence, the best way to keep track of the relevant error probabilities is to establish the statistical adequacy of $M_{\theta }\left(x\right)$ . It is important to emphasize that other forms of statistical inference, including Bayesian and Akaike-type model selection procedures, are equally vulnerable to statistical misspecification since they rely on the likelihood function $L\left(\theta ;x_{0}\right)\propto f\left(x_{0};\theta \right),\;\theta \in \Theta$ ; see Spanos [16].

In the discussion that follows, it is assumed that the invoked statistical model $M_{\theta }\left(x\right)$ is statistically adequate to avoid repetitions and digressions, but see Spanos [17] and [18] on why [a] statistical misspecification calls into question important aspects of the current replication crisis literature.

### 2.2. Frequentist Inference: Estimation

**Point estimation** revolves around an estimator, say ${\hat{\theta }}_{n}\left(X\right),$ that pinpoints (as closely as possible) $\theta^{*}$ . The clause ‘as closely as possible’ is framed in terms of certain ‘optimal’ properties stemming from the sampling distribution $f\left({\hat{\theta }}_{n}\left(x\right);\theta \right),\;x\in R_{X}^{n},$ including: unbiasedness, efficiency, sufficiency, consistency, etc.; see Casella and Berger [19]. Regrettably, the *factual reasoning,* presuming $\theta \;=\;\theta^{*},$ underlying the derivation of the relevant sampling distributions is often implicit in traditional textbook discussions, resulting in erroneous interpretations and unwarranted claims.

**Example 1** (continued). The relevant sampling distributions associated with (2), are appositely stated as:

(4) $$\begin{array}{ccc} \left[i\right]\;{\bar{X}}_{n}\overset{{\theta =\theta }^{*}}{∽}N\left(\mu_{*},\frac{\sigma_{*}^{2}}{n}\right), & \left[\mathrm{ii}\right]\;\frac{\left(n-1\right)s^{2}}{\sigma_{*}^{2}}\overset{{\theta =\theta }^{*}}{∽}\chi^{2}\left(n-1\right), & \left[\mathrm{iii}\right]\;\tau \left(X;\mu \right)\overset{{\theta =\theta }^{*}}{∽}\mathrm{St}\left(n-1\right),\\ {\bar{X}}_{n}\;=\;\frac{1}{n}\sum_{t=1}^{n}X_{t}, & s^{2}\;=\;\frac{1}{\left(n-1\right)}\sum_{t=1}^{n}{\left(X_{t}-{\bar{X}}_{n}\right)}^{2},\; & \tau \left(X;\mu \right)\;=\;\frac{\sqrt{n}\left({\bar{X}}_{n}-\mu \right)}{s},\; \end{array}$$

where $\theta^{*}:=\left(\mu^{*}and\sigma_{*}^{2}\right)$ denote the ‘true’ values of the unknown parameters, $\chi^{2}\left(n-1\right)$ denotes the chi-square distribution, and St(n−1) the Student’s t distribution, with (n−1) degrees of freedom. The problem is that without ‘ ${\theta \;=\;\theta }^{*}$ ’ the distributional results in (4) will not hold. For instance, what ensures in [i] that $E({\bar{X}}_{n})\;=\;\mu$ and $Var\left({\bar{X}}_{n}\right)\;=\;\left(\sigma^{2}/n\right)?$ The answer is the unbiasedness and full efficiency of ${\bar{X}}_{n}$ , respectively, both of which are defined at ${\theta \;=\;\theta }^{*}$ . There is no such a thing as a sampling distribution ${\bar{X}}_{n}∽N\left(\mu ,\frac{\sigma^{2}}{n}\right),$ $\forall \theta :=\left(\mu ,\sigma^{2}\right)\in R\times R_{+}$ since the NIID assumptions imply that each element of the sample X comes from a single Normal distribution with a unique mean and variance $\theta^{*}:=\left(\mu^{*},\sigma_{*}^{2}\right)$ around which all forms of statistical inference revolve. Hence, the claim by Schweder and Hjort [20] that “ $\frac{\sqrt{n}\left(\mu -{\bar{X}}_{n}\right)}{s}$ has a fixed distribution regardless of the values of the interest parameter μ and the (in this context) nuisance parameter σ… ” (p. 15), i.e.,

(5) $$\begin{array}{c} d\left(X;\mu \right)\;=\;\frac{\sqrt{n}\left(\mu -{\bar{X}}_{n}\right)}{s}∽\mathrm{St}\left(n-1\right),\;\forall \theta :=\left(\mu ,\sigma^{2}\right)\in R\times R_{+},\; \end{array}$$

makes no sense from a statistical inference perspective.

Point estimation is very important since it provides the basis for all other forms of optimal inference (CIs, testing, and prediction) via the optimality of a point estimator ${\hat{\theta }}_{n}\left(X\right).$ A point estimate ${\hat{\theta }}_{n}\left(x_{0}\right)$ , by itself, however, is considered *inadequate* for learning from data $x_{0}$ since it is unduly data specific; it ignores the relevant uncertainty stemming from the fact that $x_{0}$ constitutes a single realization (out of $\forall x\in R_{X}^{n})$ as framed by the sampling distribution, $f\left({\hat{\theta }}_{n}\left(x\right);\theta \right),\;x\in R_{X}^{n},$ of ${\hat{\theta }}_{n}\left(X\right);$ hence ${\hat{\theta }}_{n}\left(x_{0}\right)$ is often reported as ${\hat{\theta }}_{n}\left(x_{0}\right)\pm 2\sqrt{Var({\hat{\theta }}_{n})}$ .

**Interval estimation** accounts for the relevant uncertainty in terms of an error probability of ‘overlaying’ the true value $\theta^{*}$ of θ , based on $f\left({\hat{\theta }}_{n}\left(x\right);\theta \right),\;x\in R_{X}^{n}$ , in the form of the Confidence Interval (CI):

(6) $$\begin{array}{cc} P\left(L\left(X\right)\leq \theta \leq U\left(X\right);\;\theta \;=\;\theta^{*}\right) & \;=\;1-\alpha ,\; \end{array}$$

where the statistics L(X) and U(X) denote the lower and upper (random) bounds that ‘overlay’ $\theta^{*}$ with probability $\left(1-\alpha \right).$ An $\left(1-\alpha \right)$ CI is optimal when its expected length E[U(X)−L(X)] is the shortest and referred to as Uniformly Most Accurate (UMA); see Lehmann and Romano [21].

**Example 1** (continued). For (2), the two-sided optimal $\left(1-\alpha \right)$ CI for μ is based on the pivot τ(X;μ) in (4)-[iii] and takes the form:

(7) $$\begin{array}{c} \left(a\right)\;P\left[CI\left(X\right);\alpha \right]\;=\;P\left({\bar{X}}_{n}-c_{\frac{\alpha }{2}}\left(\frac{s}{\sqrt{n}}\right)\leq \mu <{\bar{X}}_{n}+c_{\frac{\alpha }{2}}\left(\frac{s}{\sqrt{n}}\right);\;\theta \;=\;\theta^{*}\right)\;=\;1-\alpha .\; \end{array}$$

The analogous one-sided optimal CIs take the form:

(8) $$\begin{array}{c} \left(b\right)\;\mathrm{Lower}:\;P\left[CI_{L}\left(X\right);\alpha \right]\;=\;P_{L}\left(\mu \geq {\bar{X}}_{n}-c_{\alpha }\left(\frac{s}{\sqrt{n}}\right)\right);\;\theta \;=\;\theta^{*})\;=\;1-\alpha ,\\ \left(c\right)\;\mathrm{Upper}:\;P\left[CI_{U}\left(X\right);\alpha \right]\;=\;P_{U}\left(\mu \leq {\bar{X}}_{n}+c_{\alpha }\left(\frac{s}{\sqrt{n}}\right);\;\theta \;=\;\theta^{*}\right)\;=\;1-\alpha . \end{array}$$

### 2.3. Frequentist Inference: Neyman-Pearson (N-P) Testing

The reasoning underlying hypothesis testing is *hypothetical,* based on prespecified values of θ, as they relate to $H_{0}$ : $\theta \in \Theta_{0}$ and $H_{1}$ : $\theta \in \Theta_{1}$ .

**Example 1** (continued). Consider testing the hypotheses of interest:

(9) $$\begin{array}{c} H_{0}:\;\mu \leq \mu_{0}\;\mathrm{vs}.\;H_{1}:\;\mu >\mu_{0},\; \end{array}$$

in the context of (2). An optimal N-P test for the hypotheses in (9) is defined in terms of a test statistic and the rejection region:

(10) $$\begin{array}{c} T_{\alpha }:=\left\{\tau \left(X\right)\;=\;\frac{\sqrt{n}\left({\bar{X}}_{n}-\mu_{0}\right)}{s},\;C_{1}\left(\alpha \right)\;=\;\left\{x:\;d\left(x\right)\;>\;c_{\alpha }\right\}\right\},\; \end{array}$$

whose error probabilities are evaluated using:

(11) $$\begin{array}{c} \left[i\right]\;\mathrm{what}\;\mathrm{if}\;\mu \;=\;\mu_{0}:\;\tau \left(X\right)\overset{\mu =\mu_{0}}{∽}\mathrm{St}\left(n-1\right),\; \end{array}$$

where St(n−1) is the Student’s t distribution with n−1 degrees of freedom, and:

(12) $$\begin{array}{c} {}[\mathrm{ii}]:\;\mathrm{what}\;\mathrm{if}\;\mu \;=\;\mu_{1},\;\tau \left(X\right)\;=\;\frac{\sqrt{n}\left({\bar{X}}_{n}-\mu_{0}\right)}{s}\overset{\mu =\mu_{1}}{∽}\;\mathrm{St}\left(\delta_{1},n-1\right),\;\forall \mu_{1}\;=\;\mu_{0}+\gamma_{1},\;\gamma_{1}\;\geq \;0,\; \end{array}$$

where St $\left(\delta_{1},n-1\right)$ is a noncentral Student’s t distribution with $\delta_{1}\;=\;\frac{\sqrt{n}\left(\mu_{1}-\mu_{0}\right)}{\sigma }.$ It is important to emphasize that (12) differs from (11) in terms of their mean, variance, and higher moments, rendering (12) non-symmetric for $\delta_{1}\neq 0$ ; see Owen [22].

The sampling distribution in (11) is used to evaluate the pre-data type I error probability and the *post-data* [ $\tau (x_{0})$ is known] *p*-value:

(13) $$\begin{array}{cc} \left(a\right)\;\alpha \;=\;P(x:\;\tau \left(X\right)\;>\;c_{\alpha };\mu \;=\;\mu_{0}), & p\left(x_{0}\right)\;=\;P\left(x:\;\tau \left(X\right)\;>\;\tau \left(x_{0}\right);\mu \;=\;\mu_{0}\right).\; \end{array}$$

The sampling distribution in (12) is used to evaluate the power of $T_{\alpha }$ :

(14) $$\begin{array}{c} \left(b\right)\;P\left(\mu_{1}\right)\;=\;P\left(x:\;\tau \left(X\right)\;>\;c_{\alpha };\mu \;=\;\mu_{1}\right),\;\forall \mu \geq \mu_{1}\;=\;\mu_{0}+\gamma_{1},\;\gamma_{1}\;\geq \;0,\; \end{array}$$

as well as the type II error probability: $\beta \left(\mu_{1}\right)\;=\;1-P\left(\mu_{1}\right),\;\forall \mu \;\geq \;\mu_{1}.$

The test $T_{\alpha }$ in (10) is optimal in the sense of being Uniformly Most Powerful (UMP), i.e., $T_{\alpha }$ is the most effective α -level test for detecting any discrepancy ( γ>0 ) of interest from $\mu \;=\;\mu_{0}$ ; see Lehmann and Romano [21].

Why prespecify α at a low threshold, such as α=0.05 ? Neyman and Pearson [23] put forward two crucial stipulations relating to the framing of $H_{0}$ : $\theta \in \Theta_{0}$ and $H_{1}$ : $\theta \in \Theta_{1},\;\Theta_{i}\subset \Theta ,\;i\;=\;0,1,$ to ensure the effectiveness of N-P testing and the informativeness of its results:

[1] $\Theta_{0}$ and $\Theta_{1}$ should form a *partition* of Θ (p. 293) to avoid $\theta^{*}\notin \left[\Theta_{0}\cup \Theta_{1}\right].$

[2] $\Theta_{0}$ and $\Theta_{1}$ should be framed in such a way so as to ensure that the type I error *is the more serious* of the two.

To provide some intuition for [2], they use the analogy with a criminal trial where to ensure [2] one should use the framing, $H_{0}$ : not guilty vs. $H_{1}$ : guilty, to render the type I error of sending an innocent person to prison, more serious than acquitting a guilty person (p. 296). Hence, prespecifying α at a small value and maximizing the power over $\forall \mu \;\geq \;\mu_{1}\;=\;\mu_{0}+\gamma_{1},\;\gamma_{1}\;\geq \;0,$ requires deliberation about the framing. A moment’s reflection suggests that stipulation [2] implies that high power is needed around the potential neighborhood of $\theta^{*}$ . Regrettably, stipulations [1]–[2] are often ignored, undermining the proper implementation and effectiveness of N-P testing; see Section 4.1.

Returning to the power of $T_{\alpha },$ the noncentrality parameter $\delta_{1}$ indicates that the power increases monotonically with $\sqrt{n}$ and $\left(\mu_{1}-\mu_{0}\right)$ and decreases with σ . This suggests that the inherent trade-off between the type I and II error probabilities in N-P testing, in conjunction with the sample size *n* and α, plays a crucial role in determining the capacity of a N-P test. This means that the selection of the significance level α should always take into account the particular *n* for data $x_{0}$ , since an uninformed choice of α can give rise to two problems.

**The small** *n* **problem**. This arises when the sample size *n* is not large enough generate any learning from data about $\theta^{*}$ since it has insufficient power to detect particular discrepancies γ of interest. To avoid underpowered tests the formula in (14) can be used *pre-data* (before $\tau (x_{0})$ is known) to evaluate the sample size *n* necessary for $T_{\alpha }$ to detect such discrepancies with high enough probability (power). That is, for a given α , there is always a small enough *n* that would accept $H_{0}$ despite the presence of a sizeable discrepancy γ≠0 of interest. This also undermines the M-S testing to evaluate the statistical adequacy of the invoked $M_{\theta }\left(x\right)$ since a small *n* will ensure that M-S tests do not have sufficient power to detect existing departures from the model assumptions; see Spanos [18].

**The large** *n* **problem**. This arises when a practitioner uses conventional significance levels, say α=0.1,0.05,0.025,0.01 , for very large sample sizes, say n> 10,000. The source of this problem is that for a given α, as *n* increases the power of a test increases and the *p*-value decreases, giving rise to over-sensitive tests. Fisher [24] explained why: “By increasing the size of the experiment [*n*], we can render it more sensitive, meaning by this that it will allow of the detection of … quantitatively smaller departures from the null hypothesis.” (pp. 21–22). Hence, for a given α, there is always a large enough *n* that would reject $H_{0}$ for any discrepancy γ≠0 (however small, say γ=0.0000001 ) from a null value $\theta \;=\;\theta_{0}$ ; see Spanos [25].

It is very important to emphasize at the outset that the pre-data testing error probabilities (type I, II, and power) are Spanos [7]:

(i) *hypothetical* and *unobservable* in principle since they revolve around $\theta^{*}$ ,

(ii) not *conditional* on values of θ∈Θ since ‘presuming $\theta \;=\;\theta_{i},\;i\;=\;0,1$ ’ constitute neither events nor random variables, and

(iii) assigned to the test procedure $T_{\alpha }$ to ‘calibrate’ its *generic* (for any $x\in R^{n}$ ) *capacity* to detect different *discrepancies* γ from $\mu \;=\;\mu_{0}$ for a prespecified α .

As mentioned above, the cornerstone of N-P testing is the in-built trade-off between the type I and II error probabilities, which Neyman and Pearson [23] addressed by prespecifying α at a low value and maximizing $P(\mu_{1}),$ $\forall \mu_{1}\;=\;\mu_{0}\;+\;\gamma_{1}\in \Theta_{1}$ , $\gamma_{1}\;\pm \;0,$ seeking an optimal test; see Lehmann and Romano [21]. The primary role of the testing error probabilities is to operationalize the notions of ‘statistically significant/insignificant’ in terms of statistical approximations relating to $\theta^{*},$ and framed in terms of the sampling distribution of a test statistic τ(X) .

This relates directly to the replication crisis since for a misspecified $M_{\theta }\left(x\right)$ one cannot keep track of the relevant error probabilities to be able to adjust them for p-hacking, data-dredging, multiple testing and cherry-picking, in light of the fact that the actual error probabilities will be different from the nominal ones; see Spanos and McGuirk [26].

### 2.4. Statistical Inference ‘Results’ vs. ‘Evidence’ for or against Inferential Claims

**Statistical results**, such as a point estimate, say $\hat{\theta }\left(x_{0}\right),$ an observed (1−α) CI, say $[L\left(x_{0}\right),U\left(x_{0}\right)],$ an effect size, a *p*-value and the accept/reject $H_{0}$ results, are not replicable in principle, in the sense that akin data do not often yield very similar numbers since they are unduly data-specific when contrasted with broader inferential claims relating to inductive generalizations stemming from such results. In particular, the accept/reject $H_{0}$ results, (i) are unduly data-specific, (ii) are too coarse to provide informative enough evidence relating to $\theta^{*}$ , and (iii) depend crucially on the particular statistical context:

(15) $$\begin{array}{c} \left(i\right)M_{\theta }\left(x\right),\;\left(\mathrm{ii}\right)H_{0}:\theta \in \Theta_{0}\mathrm{vs}.H_{1}:\theta \in \Theta_{1},\left(\mathrm{iii}\right)T_{\alpha }:=\left\{d\left(X\right),C_{1}\left(\alpha \right)\right\},\left(\mathrm{iv}\right)\mathrm{data}x_{0},\; \end{array}$$

which includes the statistical adequacy of $M_{\theta }\left(x\right)$ as well as the sample size *n*.

**Example 1** (continued). It is often erroneously presumed that the optimality of the point estimators, $\hat{\mu }\left(X\right)\;=\;{\bar{X}}_{n}\;=\;\frac{1}{n}\sum_{t=1}^{n}X_{t},\;s^{2}\left(X\right)\;=\;\frac{1}{\left(n-1\right)}\sum_{t=1}^{n}{\left(X_{t}-{\bar{X}}_{n}\right)}^{2}$ , can justify the following inferential claims for the particular data $x_{0}$ when *n* is large enough.

(a) The point estimates $\hat{\mu }\left(x_{0}\right)\;=\;{\bar{x}}_{n}$ and $s^{2}\left(x_{0}\right)\;=\;s_{n}^{2},$ based on data $x_{0},$ ‘approximate closely’ (≃) the true parameter values $\mu^{*}$ and $\sigma_{*}^{2},$ i.e.,

(16) $$\begin{array}{c} \hat{\mu }\left(x_{0}\right)≃\mu^{*},\mathrm{and}s^{2}\left(x_{0}\right)≃\sigma_{*}^{2},\mathrm{for}\;n\;\mathrm{large}\;\mathrm{enough}.\; \end{array}$$

Invoking limit theorems, such as strong consistency, will not alleviate the problem since, as argued by Le Cam [27], p. xiv: “… limit theorems ‘as *n* tends to infinity’ are logically devoid of content about what happens at any particular *n*.” The inferential claims in (16) are unwarranted since $\hat{\mu }\left(x_{0}\right)$ and $s^{2}\left(x_{0}\right)$ ignore the relevant uncertainty associated with their representing a single point, $X\;=\;x_{0},$ from the relevant sampling distributions: $\;f\left(\hat{\mu }\left(x\right);\theta \right),\;f\left(s^{2}\left(x\right);\theta \right),\;\forall x\in R^{n};$ see Spanos [7].

(b) The inferential claim associated with an $\left(1-\alpha \right)$ optimal CI for μ in (7) relates to CI (X;μ) overlaying $\mu^{*}$ with probability (1−α), but its optimality does not justify the claim that the observed CI:

(17) $$\begin{array}{c} CI\left(x_{0}\right)\;=\;[{\bar{x}}_{n}-c_{\frac{\alpha }{2}}\left(s\left(x_{0}\right)/\sqrt{n}\right),\;{\bar{x}}_{n}+c_{\frac{\alpha }{2}}\left(s\left(x_{0}\right)\right],\; \end{array}$$

overlays $\mu^{*}$ with probability (1−α). As argued by Neyman [28]: “… valid probability statements about random variables usually cease to be valid if the random variables are replaced by their particular values.” (p. 288). In terms of the underlying factual reasoning, post-data $x_{0}$ has been revealed but it is unknowable whether $\mu^{*}$ is within or outside (17); see Spanos [29]. Indeed, one can make a case that the widely held impression that an effect size ( [30]) provides more reliable information about the ‘scientific effect’ than *p*-values and observed CIs stems from the unwarranted inferential claim in (16), i.e., an optimal estimator $\hat{\theta }\left(X\right)$ of θ justifies the inferential claim $\hat{\theta }\left(x_{0}\right)≃$ $\theta^{*}$ for *n* large enough.

(c) The N-P testing ‘accept $H_{0}$ ’ with a large *p*-value, and rejecting $H_{0}$ with a small *p*-value, do not entail evidence for $H_{0}$ and $H_{1}$ , respectively, since such evidential interpretations are fallacious; see Mayo and Spanos [14].

## 3. Post-Data Severity Evaluation of Testing Results

### 3.1. Accept/Reject $H_{0}$ Results vs. Evidence for or against Inferential Claims

Bridging the gap between the binary accept/reject $H_{0}$ results and learning from data $x_{0}$ about $\theta^{*}$ using statistical approximations framed in terms of the sampling distribution of a test statistic τ(X) , has been confounding frequentist testing. Mayo and Spanos [31] proposed the post-data severity (SEV) evaluation of the accept/reject $H_{0}$ results as a way to convert them into evidence for germane inferential claims. The SEV differs from other attempts to address this issue in so far as:

(i) The SEV evaluation constitutes a principled argument framed in terms of a germane inferential claim relating to $\theta^{*}$ (learning from data $x_{0}$ ).

(ii) The SEV evaluation is guided by the sign and magnitude of the observed test statistic, $\tau (x_{0}),$ and not by the prespecified significance level α ; see Spanos [32].

(iii) The SEV evaluation accounts fully for the relevant statistical context in (15).

(iv) Its germane inferential claim, in the form of the discrepancy from the null value, is warranted with high probability with $x_{0}$ and $T_{\alpha }$ when all the different ways it can be false have been adequately probed and forfended (Mayo [33]).

The most crucial way to forfend a false accept/reject $H_{0}$ result is to ensure that $M_{\theta }\left(x\right)$ is statistically adequate for data $x_{0}$ , before any inferences are drawn. This is because the discrepancies induced by invalid probabilistic assumptions will render impossible the task of controlling (keeping track of) the relevant error probabilities in terms of which N-P tests are framed. Hence, for the discussion that follows it is assumed that $M_{\theta }\left(x\right)$ is statistically adequate for the particular data $x_{0}$ .

**Example 2**. Consider the simple Bernoulli (Ber) model:

(18) $$\begin{array}{cc} M_{\theta }\left(x\right): & X_{k}∽\mathrm{BerIID}\left(\theta ,\theta \left(1-\theta \right)\right),\;\;x_{k}\;=\;0,1,\;0\;<\;\theta \;<\;1,\;k\in N,\; \end{array}$$

where $\theta \;=\;E\left(X_{k}\right)\;=\;P\left(X_{k}\;=\;1\right),\;P\left(X_{k}\;=\;0\right)\;=\;\left(1-\theta \right)$ . Let the hypotheses of interest be:

(19) $$\begin{array}{c} H_{0}:\;\theta \leq \theta_{0}\;\mathrm{vs}.\;H_{1}:\;\theta >\theta_{0},\;\theta_{0}\;=\;0.5,\; \end{array}$$

in the context of (18). It can be shown that the t-type test:

(20) $$\begin{array}{c} T_{\alpha }^{>}:=\{d\left(X\right)\;=\;\frac{\sqrt{n}\left({\bar{X}}_{n}-\theta_{0}\right)}{\sqrt{\theta_{0}\left(1-\theta_{0}\right)}},\;C_{1}\left(\alpha \right)\;=\;\left\{x:d\left(x\right)\;>\;c_{\alpha }\right\},\; \end{array}$$

where ${\bar{X}}_{n}\;=\;\frac{1}{n}\sum_{t=1}^{n}X_{t},$ is Uniformly Most Powerful (UMP); see Lehmann and Romano [21]. The sampling distribution of d(X), evaluated under $H_{0}$ (hypothetical), is:

(21) $$\begin{array}{c} d\left(X\right)\;=\;\frac{\sqrt{n}\left({\bar{X}}_{n}-\theta_{0}\right)}{\sqrt{\theta_{0}\left(1-\theta_{0}\right)}}\overset{\theta =\theta_{0}}{∽}\mathrm{Bin}\left(0,1;n\right)≃N\left(0,1\right).\; \end{array}$$

For n≥40 the ‘standardized’ Binomial distribution, $\mathrm{Bin}\left(0,1;n\right),$ can be approximated (≃) by the N (0,1). The latter can be used to evaluate the type I error probability and the *p*-value:

(22) $$\begin{array}{cc} \alpha \;=\;P(d\left(X\right)\;>\;c_{\alpha };\;\theta \;=\;\theta_{0}), & p\left(x_{0}\right)\;=\;P\left(d\left(X\right)\;>\;d\left(x_{0}\right);\;\theta \;=\;\theta_{0}\right). \end{array}$$

The sampling distribution of d(X) evaluated under $H_{1}$ (hypothetical) is:

(23) $$\begin{array}{c} d\left(X\right)\;=\;\frac{\sqrt{n}\left({\bar{X}}_{n}-\theta_{0}\right)}{\sqrt{\theta_{0}\left(1-\theta_{0}\right)}}\overset{\theta =\theta_{1}}{∽}\mathrm{Bin}\left(\delta \left(\theta_{1}\right),\sqrt{V(\theta_{1})};n\right),\;\mathrm{for}\;\theta_{1}\;=\;\theta_{0}\;+\;\gamma_{1},\;\gamma_{1}\geq 0,\\ \delta \left(\theta_{1}\right)\;=\;\frac{\sqrt{n}\left(\theta_{1}-\theta_{0}\right)}{\sqrt{\theta_{0}\left(1-\theta_{0}\right)}},\;V\left(\theta_{1}\right)\;=\;\frac{\theta_{1}\left(1-\theta_{1}\right)}{\theta_{0}\left(1-\theta_{0}\right)},\; \end{array}$$

whose tail area probabilities can be approximated using:

(24) $$\begin{array}{c} {\left(\sqrt{V(\theta_{1})}\right)}^{-1}\left[d\left(X\right)-\delta \left(\theta_{1}\right)\right]\overset{\theta \;=\;\theta_{1}}{∽}\mathrm{Bin}\left(0,1;n\right)≃N\left(0,1\right). \end{array}$$

(24) is used to derive the type II error probability and the power of the test $T_{\alpha }^{>}$ in (20) which increases monotonically with $\sqrt{n}$ and $\left(\mu_{1}-\mu_{0}\right)$ and decreases with $V(\theta_{1})$ .

The post-data severity (SEV) evaluation transforms the ‘accept/reject $H_{0}$ results’ into ‘evidence’ for or against germane inferential claims framed in terms of θ . The post-data severity evaluation is defined as follows:

A hypothesis *H* ( $H_{0}$ or $H_{1}$ ) *passes a severe test* $T_{\alpha }$ with data $x_{0}$ if:

(C-1) $x_{0}$ accords with *H*, and

(C-2) with very high probability, test $T_{\alpha }$ would have produced a result that ‘accords less well’ with *H* than $x_{0}$ does, if *H* were false; see Mayo and Spanos [31,34].

**Example 2** (continued). Consider data $x_{0}$ referring to newborns during 1995 in Cyprus, 5152 boys $\left(X\;=\;1\right)$ and 4717 girls $\left(X\;=\;0\right)$ . In this case, there is no reason to question the validity of the IID probabilistic assumptions since nature ensures their validity when such data are collected over a sufficiently long period of time in a particular locality. Applying the optimal test $T_{\alpha }^{>}$ in (20) with α=0.01 (large *n*) yields:

$$\begin{array}{c} d\left(x_{0}\right)\;=\;\frac{\sqrt{9869}\left(\frac{5152}{9869}-0.5\right)}{\sqrt{0.5(1-0.5)}}\;=\;4.379,\mathrm{indicating}\;‘\mathrm{reject}\;H_{0}^{\prime }\;\mathrm{with}\;p_{>}\left(x_{0}\right)\;=\;0.000006.\; \end{array}$$

Broadly speaking, this result indicates that the ‘true’ value $\theta^{*}$ of θ lies within the interval $\left(0.5,1\right)$ which is too coarse to engender any learning about $\theta^{*}.$

The post-data severity outputs a germane evidential claim that revolves around a discrepancy $\gamma^{‡}$ warranted by test $T_{\alpha }^{>}$ and data $x_{0}$ with high probability. In contrast to pre-data testing error probabilities (type I, II, and power), severity is a *post-data error probability* that uses additional information in the form of the sign and magnitude of $d(x_{0})$ , but shares with the former the underlying *hypothetical reasoning*: presuming that $\theta \;=\;\theta_{1},\;\forall \theta_{1}\in \Theta_{1}$ .

Given that $d\left(x_{0}\right)\;=\;4.379>c_{\alpha }$ ,

[C-1] $x_{0}$ accords with $H_{1}$ and since $d(x_{0})>0$ , the relevant inferential claim takes the form $\theta \;>\;\theta_{1}\;=\;\theta_{0}+\gamma_{1},\;\gamma_{1}\;\geq \;0.$

**Figure 1:**
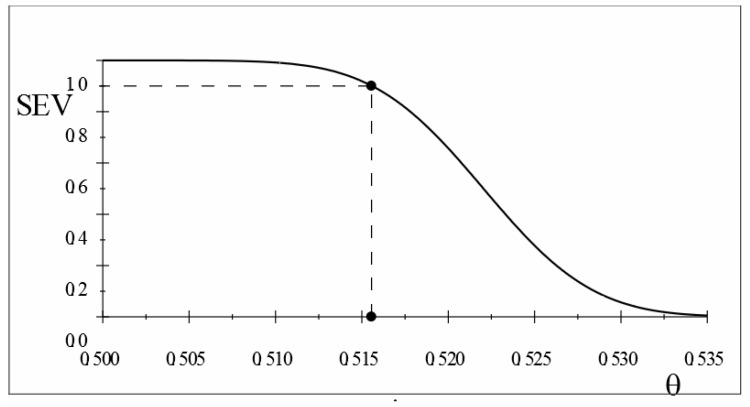
Severity curve for test $T_{\alpha }^{>}$ and data $x_{0}$ .

[C-2] revolves around the event: “outcomes x that accord less well with $\theta \;>\;\theta_{1}$ than $x_{0}$ does”, i.e., event {x : $d\left(x\right)\leq d\left(x_{0}\right)\},\;\forall x\in {\left\{0,1\right\}}^{n},$ and its probability:

(25) $$\begin{array}{c} SEV\left(T_{\alpha }^{>};\theta \;>\;\theta_{1}\right)\;=\;P\left(d\left(X\right)\leq d\left(x_{0}\right);\;\theta \;=\;\theta_{1}\right),\;\forall \theta_{1}\in \Theta_{1}\;=\;\left(0.5,1\right), \end{array}$$

stemming from (23). This severity curve $\forall \theta_{1}\in \Theta_{1}\;=\;\left(0.5,0.53\right)$ is depicted in Figure 1.

In the case of ‘reject $H_{0}$ ’ the objective is to evaluate the largest $\theta_{1}\;=\;\theta_{0}+\gamma_{1}$ such that any θ less than $\theta_{1}$ would very probably, at least 0.9, have resulted in a smaller observed difference, warranted by test $T_{\alpha }^{>}$ and data $x_{0}$ :

$$\begin{array}{c} \max_{\theta_{1}\in \left(0.5,1\right)}SEV\left(T_{\alpha }^{>};x_{0};\theta \;>\;\theta_{1}\right)\;=\;0.9\to \gamma_{1}^{‡}\leq 0.01557\; \end{array}$$

Like all error probabilities, the SEV evaluation is always attached to the procedure itself as it pertains to the inferential claim $\theta \;>\;\theta_{1}$ . The inferential claim $\gamma_{1}^{‡}\leq 0.01557$ warranted with probability 0.9 , however, can be ‘informally’ interpreted as evidence for a germane neighborhood of $\theta^{*}$ , $\theta_{1}\;=\;0.51557\pm \varepsilon ,$ for some ε≥0 , arising from the SEV evaluation narrowing down the coarse $\theta^{*}\in \left(0.5,1\right)$ associated with the ‘reject $H_{0}$ ’. This narrowing down of the potential neighborhood of $\theta^{*}$ enhances learning from data.

It is also important to emphasize that the SEV evaluation of the inferential claim $\theta \;>\;\theta_{1}\;=\;\theta_{0}+\gamma_{1}$ , $\gamma_{1}\;\geq \;0$ with discrepancy $\gamma_{1}\;=\;0.0223,$ based on ${\bar{x}}_{n}\;=\;0.5223$ , gives rise to $SEV(T_{\alpha }^{>};x_{0};\theta \;>\;0.5223)\;=\;0.5,$ which implies that there is no evidence for $\theta_{1}\;\leq \;0.5223.$ More generally, the SEV evaluation will invariably provide evidence *against* the inferential claim $\theta_{1}\leq {\bar{x}}_{n}.$ Hence, the importance of distinguishing between ‘statistical results’, such as ${\bar{x}}_{n}\;=\;0.5223$ , and ‘evidence’ for or against inferential claims relating to ${\bar{x}}_{n}$ .

What is the *nature of evidence* the post-data severity (SEV) gives rise to? Since the objective of inference is to learn from data about phenomena of interest via learning about $\theta^{*}$ , the evidence from the SEV comes in the form of an inferential claim that revolves around the discrepancy $\gamma_{1}$ warranted by the particular data and test with high enough probability, pinpointing the neighborhood of $\theta^{*}$ as closely as possible. In the above case, the warranted discrepancy is $\gamma_{1}^{‡}\leq 0.01557,$ or equivalently, $\theta^{*}\;\leq \;0.51557$ , with probability 0.9. Although all probabilities are assigned to the inference procedure itself as it relates to the inferential claim $\theta \;>\;\theta_{1}\;=\;\theta_{0}+\gamma_{1},\;\gamma_{1}\;>\;0,$ the SEV evaluation can be viewed intuitively as narrowing the coarse reject $H_{0}$ result entailing $\theta^{*}\in \left(0.5,1\right)$ down to $\theta^{*}\in \left(0.512,0.5156\right).$

The most important attributes of the SEV evaluation are:

[i] It is a *post-data* error probability stemming from *hypothetical* reasoning that takes into account the statistical context in (15) and is guided by $d(x_{0})≷0$ . 

[ii] Its evaluation is invariably based on a discrepancy ${\left(\sqrt{V(\theta_{1})}\right)}^{-1}\left[d\left(x_{0}\right)-\delta \left(\theta_{1}\right)\right]$ relating to the noncentral distribution in (24), where $d(x_{0})$ and $\delta (\theta_{1})$ use the same *n* to output the warranted discrepancy $\gamma_{1}\;=\;\left(\theta_{1}-\theta_{0}\right)\geq 0$ .

### 3.2. The Robustness of the Post-Data Severity Evaluation

To exemplify the robustness of the SEV evaluation with respect to changing $\theta_{0}$ , consider replacing $\theta_{0}\;=\;0.5$ in (19) with the Nicolas Bernoulli value $\theta_{0}\;=\;\left(18/35\right)≃0.5143$ .

**Example 2** (continued). Applying the same test $T_{\alpha }^{>}$ yields

$$\begin{array}{c} d_{B}\left(x_{0}\right)\;=\;\sqrt{9869}\left(\frac{5152}{9869}-0.5143\right)/\sqrt{0.5143(1-0.5143)}\;=\;1.541,\mathrm{indicating}\;‘\mathrm{accept}H_{0}^{\prime },\; \end{array}$$

with $p(x_{0})\;=\;0.062$ . Is this ‘accept $H_{0}$ ’ result at odds with the previous ‘reject $H_{0}$ ’ result? In light of $d_{B}\left(x_{0}\right)\;>\;0,$ the relevant inferential claim is identical to the case with $\theta_{0}\;=\;0.5,$ $\theta \;>\;\theta_{1}\;=\;\theta_{0}+\gamma_{1},$ $\gamma_{1}\;>\;0,$ as it relates to the event {x : $d\left(x\right)\leq d\left(x_{0}\right)\},\;\forall x\in {\left\{0,1\right\}}^{n}.$ Thus, the severity curve $SEV_{B}\left(T_{\alpha }^{>};\theta \;>\;\theta_{1}\right)$ is identical $\forall \theta_{1}\in \left(0.5,1\right)$ to one in Figure 1, but now defined with respect to $\theta_{0}\;=\;\frac{18}{35}\;=\;0.5143$ , i.e.,

$$\begin{array}{c} \min_{\theta_{1}\in \left(0.5,1\right)}SEV\left(T_{\alpha }^{>};x_{0};\theta \;>\;\theta_{1}\right)\;=\;0.9\to \gamma_{1}^{‡}\leq 0.00127,\; \end{array}$$

as shown in Table 1, a feature of a sound account of statistical evidence.

### 3.3. Post-Data Severity and the Replicability of Evidence

The post-data severity evaluation of the ‘accept/reject $H_{0}$ ’ results can also provide a more robust way to evaluate the replicability of empirical evidence based on comparing the discrepancies γ from the null value, $\theta \;=\;\theta_{0}$ warranted with similar data with high enough severity. To illustrate this, consider the following example that uses similar data $x_{1}$ from a different country more than three centuries apart.

**Example 3.** Data $x_{1}$ refer to newborns during 1668 in London (England), 6073 boys, 5560 girls, n= 11,633; see Arbuthnot [35]. The optimal test in (20) yields $d(x_{1})\;=\;4.756,$ with $p(x_{1})\;=\;0.0000001,$ rejecting $H_{0}$ : θ≤0.5. This result is almost identical to the result with data from Cyprus for 1995, but the question is whether the latter can be viewed as a successful replication with trustworthy evidence.

Evaluating the post-data severity with the same probability $SEV(T_{\alpha }^{>};x_{1};\theta \;>\;\theta_{1})\;=\;0.9$ , the warranted discrepancy from $\theta_{0}\;=\;0.5$ by test $T_{\alpha }^{>}$ and data $x_{1}$ is $\gamma_{1}^{‡}\leq 0.01516$ , which is very close to $\gamma_{0}^{‡}\leq 0.01557$ ; a fourth decimal difference.

**Figure 2:**
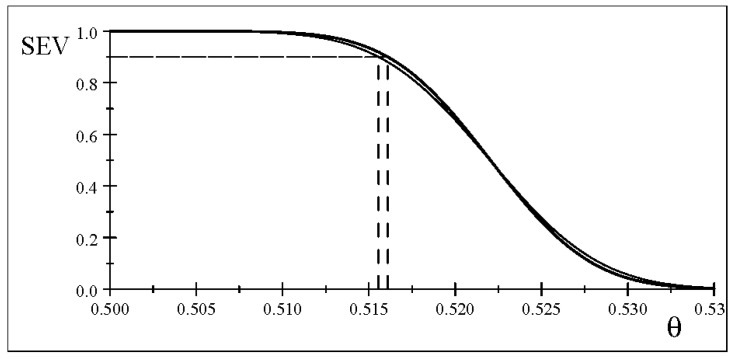
Severity curves for data $x_{i},\;i\;=\;0,1$ .

As shown in Figure 2, the severity curves for data $x_{0}$ and $x_{1}$ almost coincide. This suggests that, for a statistically adequate $M_{\theta }\left(x\right)$ , the post-data severity could provide a more robust measure of replicability associated with trustworthy evidence, than point estimates, effect sizes, observed CIs, or *p*-values. Indeed, it can be argued that the warranted discrepancy γ with high probability provides a much more robust testing-based effect size for the scientific effect; see Spanos [7].

### 3.4. Statistical vs. Substantive Significance

The post-data severity evaluation can also address this problem by relating the discrepancy $\gamma^{‡}$ from $\theta_{0}$ ( $\theta_{1}\;=\;\theta_{0}\pm \gamma^{‡}$ ) warranted by test $T_{\alpha }$ and data $x_{0}$ with high probability, to the substantively (human biology) determined value $\varphi^{⧫}$ . For that, one needs to supplement the statistical information in data $x_{0}$ with reliable substantive subject matter information to evaluate the ‘scientific effect’.

**Example 2** (continued). Human biology informs us that the *substantive value* for the ratio of boys to all newborns is $\varphi^{⧫}≃0.5122$ ; see Hardy [36]. Comparing $\varphi^{⧫}$ with the severity-based warranted discrepancy, $\gamma_{1}^{‡}\;\leq \;0.01557$ ( $\theta_{1}\;\leq \;0.51557$ ) indicates that the statistically determined $\gamma_{1}^{‡}$ entails the substantive significance, since $\varphi^{⧫}≃0.5122<0.51557$ . Hence, the statistical value also implies substantive significance.

### 3.5. Post-Data Severity and the Large n Problem

To alleviate the large *n* problem, some textbooks in statistics advise practitioners to keep reducing α as *n* increases beyond n>200; see Lehmann and Romano [21]. A less arbitrary method is to agree that, say, α=0.05 for n=100, seems a reasonable standard, and then modify Good’s [37] standardization of the *p*-values into thresholds α(n) using the formula: $\alpha \left(n\right)=0.05/\sqrt{n/100},\;n\;\geq \;50,$ as shown in Table 2. This standardization is also ad hoc since (i) it depends on an agreed standard, (ii) the simple scaling, but (iii) for $n\;\geq \;{1\;\times \;10}^{8}$ the implied thresholds are tiny.

The post-data severity evaluation of the accept/reject $H_{0}$ results can be used to shed light on the link between α and n. Let us return to example 1 and assume that n=1000 is large enough (a) to establish the statistical adequacy of the simple Bernoulli model in (18), (b) to avoid the small *n* problem, and (c) to provide a reliable enough estimate $\hat{\theta }\left(x_{0}\right)\;=\;{\bar{x}}_{n}$ for θ . There are two possible scenarios one can consider.

Scenario 1 assumes that all different values of n≥1000 yield the same observed value of the test statistic $d(x_{0})$ . This scenario has been explored in the context of the SEV evaluation by Mayo and Spanos [31].

Scenario 2 assumes that as *n* increases beyond n=1000 the changes in the estimate $\hat{\theta }\left(x_{0}\right)\;=\;{\bar{x}}_{n}$ will be ‘relatively small’ to render $\left[\left(\hat{\theta }\left(x_{0}\right)-\theta_{0}\right)/\sqrt{\theta_{0}\left(1-\theta_{0}\right)}\right]$ approximately constant when the IID assumptions are valid for $x_{n}$ .

To explore scenario 2, let us return to example 2, related to testing $H_{0}$ : $\theta \leq \theta_{0}$ vs. $H_{1}$ : $\theta \;>\;\theta_{0},\;\theta_{0}\;=\;0.5,$ in the context of the simple Bernoulli model in (18), using data on newborns in Cyprus during 1995, 5152 (male) and 4717 (female). Particular values for *n* and $p_{n}\left(x_{0}\right)$ are given in Table 3, indicating clearly that for n> 20,000 the *p*-value goes to zero very fast, and thus, the thresholds α(n) needed to counter the increase in *n* will be tiny.

**Figure 3:**
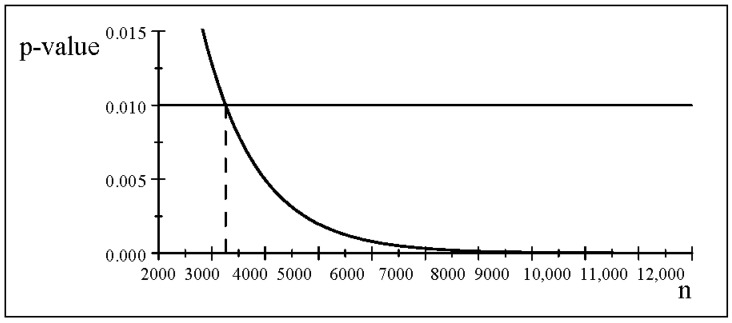
The *p*-value for α=0.01 and different *n*.

Figure 3 shows the *p*-value for different values of *n*, indicating that for n≥3256 the null hypothesis will be rejected, but for smaller *n* will be accepted. This indicates clearly that for a given α the accept/reject results are highly vulnerable to abuse stemming from manipulating the sample size to obtain the desired result. This abuse can be addressed by the SEV evaluation for such results.

**Figure 4:**
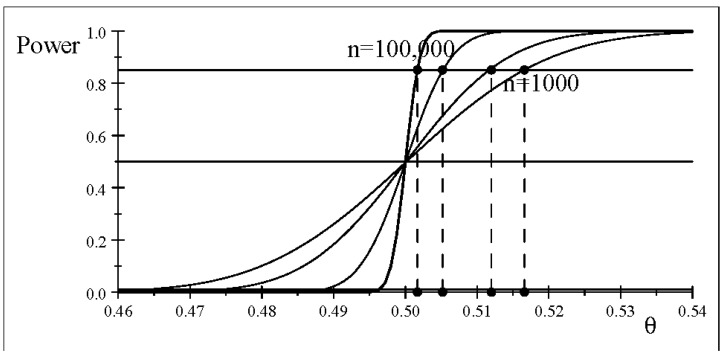
The power curves for α=0.01 and different *n*.

The other side of the large *n* coin relates to the increase in power for a given α=0.01 as *n* increases. Figure 4 shows that the power curve becomes steeper and steeper as *n* increases, reflecting the detection of smaller and smaller discrepancies from $\theta_{0}\;=\;0.5$ with probability 0.85 . This stems from the fact that the power of the test in (20) is evaluated using the difference between a fixed $c_{\alpha }$ and $\delta (\theta_{1})$ , which increases with $\sqrt{n}$ , based on (24).

The above numerical examples relating to test (20) under scenario 2 suggest that rules of thumb relating to decreasing α as *n* increases, in an attempt to meliorate potentially spurious results, can be useful in tempering the trade-off between the type I and II error probabilities. They do not, however, address the large *n* problem, since they are ad hoc and their standardized thresholds decrease to zero beyond n= 100,000.

The post-data severity evaluation (SEV) of the accept/reject $H_{0}$ results constitutes a principled argument that addresses the large *n* problem by ensuring that the same *n* is used in both terms $d_{n}\left(x_{n}\right)$ and $\delta (\theta_{1})$ when the warranted discrepancy $\gamma_{1}$ is evaluated based on

(26) $$\begin{array}{c} {\left(\sqrt{V(\theta_{1})}\right)}^{-1}\left[d_{n}\left(x_{n}\right)-\delta \left(\theta_{1}\right)\right],\;\forall \theta_{1}\in \left(0.5,1\right),\; \end{array}$$

using (24), in contrast to the power, which replaces $d_{n}\left(x_{n}\right)$ with $c_{\alpha }$ in (26). To illustrate this argument, Table 4 reports the SEV evaluations using scenario 2, where $\left(0.52204-0.5\right)/\sqrt{0.5(1-0.5)}$ is retained and *n* is allowed to vary below and above the original n=9869 for values that give rise to reject $H_{0}$ . The numbers indicate that for a given $SEV(\theta \;>\;\theta_{1};n)\;=\;0.9$ , as *n* increases, the warranted discrepancy $\gamma_{n}^{‡}$ increases, or equivalently the SEV(θ>0.51557;n) for $\gamma_{1}^{‡}\;=\;0.01557$ increases with n.

**Figure 5:**
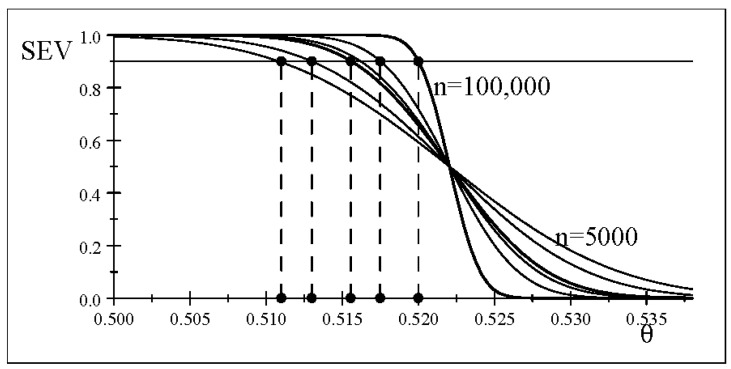
Severity curves for test $T_{\alpha }^{>}$ and data $x_{0}$ .

The severity curves $SEV(T_{\alpha }^{>};\theta \;>\;\theta_{1};n)$ for the different *n* in Table 4 are shown in Figure 5, with the original n=9869 and $n\;=\;1\;\times \;10^{5}$ in heavy lines. The curves confirm the results in Table 4, and provide additional details, indicating the need to increase the benchmark of how high the probability $SEV(\theta \;>\;\theta_{1};n)$ should be to counter-balance the increase in *n*; hence the use of 0.9 for example 2 ( n=9869 ) and 3 ( n= 11,633), and 0.7 ( n=20 ) for example 4.

## 4. Post-Data Severity and the Remedies Proposed by the Replication Literature

### 4.1. The Alleged Arbitrariness in Framing $H_{0}$ and $H_{1}$

The issue of the framing of $H_{0}$ and $H_{1}$ in N-P testing has been widely misconstrued by the replication crisis literature, questioning its coherence and blaming the accept/reject $H_{0}$ results and the *p*-value, as providing misleading evidence and often non-replicable results. Contrary to that, in addition to Neyman and Pearson [23] warning against misinterpreting ‘accept $H_{0}$ ’ as evidence for $H_{0}$ and ‘reject $H_{0}$ ’ as evidence for $H_{1},$ they also put forward two stipulations (1–2) (Section 2.3) relating to the framing of $H_{0}$ and $H_{1},$ whose objective is to ensure the effectiveness of N-P testing and the informativeness of the ensuing results. The following example illustrates how a ‘nominally’ optimal test can be transformed into a wild goose chase when (1–2) are ignored.

**Example 4**. In an attempt to demonstrate the ineptitude of the *p*-value as it compares to Bayesian testing, Berger [38] p. 4, put forward an example of testing:

(27) $$\begin{array}{c} H_{0}:\theta \;=\;0.5\mathrm{vs}.H_{1}:\theta \;>\;0.5,\; \end{array}$$

in the context of the simple Bernoulli model in (18), with $\alpha \;=\;0.05,\;c_{\alpha }\;=\;1.645,$ n=20 , and $\hat{\theta }\left(x_{0}\right)\;=\;{\bar{x}}_{n}\;=\;0.2$ . Applying the UMP test $T_{\alpha }^{>}$ yields: $d(x_{0})\;=\;-2.683,$ with a *p*-value $p_{>}\left(x_{0}\right)\;=\;0.996$ , indicating ‘accept $H_{0}$ ’. Berger avails this “ridiculous”result to make a case for Bayesian statistics: “A sensible Bayesian analysis suggests that the evidence indeed favors $H_{0}$ , but only by a factor of roughly 5 to 1.” (p. 4). Viewing this result in the context of the N-P stipulations (1–2) (Section 2.3) reveals that the real source of this absurd result is likely to be the framing in (27) since it flouts both stipulations. Assuming the statistical adequacy of the invoked $M_{\theta }\left(x\right)$ in (18), $\hat{\theta }\left(x_{0}\right)\;=\;0.2$ gives a broad indication of the potential neighborhood of $\theta^{*}.$ In contrast, the framing in (27) ensures that the test $T_{\alpha }^{>}$ has no power to detect any discrepancies around $\theta_{1}\;=\;\hat{\theta }\left(x_{0}\right)\pm \epsilon ,\;\epsilon \;>\;0$ , since the implicit power is $P(\theta_{1}\;=\;0.2)\;=\;0.00000003,$ confirming that $p_{>}\left(x_{0}\right)\;=\;0.996$ is the result of ignoring stipulations (1–2).

How can one avoid such abuses of N-P testing and secure trustworthy evidence? When there is no reliable information about the potential neighborhood around $\theta^{*}$ , one should always use the two-sided framing

$$\begin{array}{c} H_{0}:\;\theta \;=\;0.5\;\mathrm{vs}.\;H_{1}:\;\theta \neq 0.5,\; \end{array}$$

that accords with the N-P stipulations (1–2). Applying the UMP unbiased test

$$\begin{array}{c} T_{\alpha }^{\neq }:=\left[d\left(X\right)\;=\;\frac{\sqrt{n}\left({\bar{X}}_{n}-\theta_{0}\right)}{\sqrt{\theta_{0}\left(1-\theta_{0}\right)}},\;C_{1}\left(\alpha \right)\;=\;\left\{x:\left|d(x)\right|\;>\;c_{\frac{\alpha }{2}}\right\}\right] \end{array}$$

(Lehmann and Romano [21]), reject $H_{0}$ with $p_{\neq }\left(x_{0}\right)\;=\;0.0073$ , with $P_{\neq }\left(\theta_{1}\;=\;0.2\pm \epsilon \right)\;\geq \;0.94$ for ϵ=0.1 . An even stronger rejection, with $p_{<}\left(x_{0}\right)\;=\;0.004$ , will result by testing the hypotheses

(28) $$\begin{array}{c} H_{0}:\theta \;>\;0.5\mathrm{vs}.H_{1}:\theta \leq 0.5,\; \end{array}$$

using the UMP test $T_{\alpha }^{<}:=\{d\left(X\right)=\frac{\sqrt{n}\left({\bar{X}}_{n}-\theta_{0}\right)}{\sqrt{\theta_{0}\left(1-\theta_{0}\right)}}\},\;C_{1}\left(\alpha \right)\;=\;\{x:d\left(x\right)<c_{\alpha }\}.$

**Figure 6:**
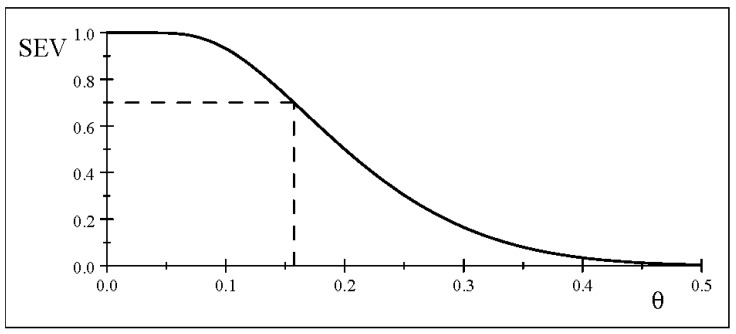
Severity curve for test $T_{\alpha }^{<}$ and data $\hat{\theta }\left(x_{0}\right)\;=\;0.2$ .

Applying the SEV evaluation to the result ‘reject $H_{0}$ ’, with $d(x_{0})\;=\;-2.683$ based on (28), it is clear that the sign and magnitude of $d(x_{0})$ indicate that the relevant inferential claim is $\theta \;>\;\theta_{1}\;=\;0.5-\gamma_{1},$ $\gamma_{1}\;>\;0.$ (C-2) implies that the relevant event is {x : $d\left(x\right)\;>\;d\left(x_{0}\right)\},\;\forall x\in {\left\{0,1\right\}}^{n},$ to infer the warranted discrepancy with high probability, say, 0.7 ( n=20 ):

$$\begin{array}{c} \max_{\theta_{1}\in \left(0,0.5\right)}SEV\left(T_{\alpha }^{>};\theta \;>\;\theta_{1}\right)\;=\;P\left(d\left(X\right)<d\left(x_{0}\right);\;\theta \;=\;\theta_{1}\right)\;=\;0.7,\;\forall \theta_{1}\in \Theta_{1}\;=\;\left(0,0.5\right),\; \end{array}$$

which yields $\gamma^{‡}\leq 0.343\;\left(\theta \leq 0.157\right),$ with the severity curve in Figure 6.

Note: the severity curve for the two-sided test would have been identical to that in Figure 6, since $d(x_{0})\;=\;-2.683$ determines the direction of departure, irrespective of $\pm c_{\frac{\alpha }{2}}.$

Applying the post-data severity evaluation for discrepancies γ>−0.03 ( $\theta_{1}>0.47$ ) gives $SEV(T_{\alpha }^{>};\theta \;>\;\theta_{1}\;=\;0.47)\leq 0.008,$ which is the strongest possible evidence against $\theta_{0}\;=\;0.5$ , repudiating the Bayesian posterior odds of 5 to 1 *for* $\theta_{0}\;=\;0.5$ .

A potential counter-argument to the above discussion, claiming that the estimate ${\bar{x}}_{n}\;=\;0.2$ could be the result of $x_{0}$ a *bad draw*, is not a well-grounded argument, since misspecification testing of the invoked $M_{\theta }\left(x\right)$ would reveal whether $x_{0}$ is atypical, i.e., a bad draw from the sample X . Recall that the adequacy of $M_{\theta }\left(x\right)$ ensures that $x_{0}$ is a ‘typical’ realization thereof. It is worth mentioning, however, that example 4 with n=20 could be vulnerable to the small *n* problem discussed in Section 2.3.

### 4.2. The Call for Redefining Statistical Significance

In light of the above discussion on the large *n* problem, the call by Benjamin et al. [6] “For fields where the threshold for defining statistical significance for new discoveries is P<0.05 , we propose a change to P<0.005 .” (p. 6) seems visceral! It brushes aside the inherent trade-off between the type I and II error probabilities and the implied inverse relationship between the sample size *n* and the appropriate α to avoid the large/small *n* problems; see Section 2.3. The main argument used by Benjamin et al. [6] is that empirical evidence from large-scale replications indicates that studies with $p(x_{0})\;<\;0.005$ are more likely to be replicable than those based on $p(x_{0})\;<\;0.05$ .

When this claim is viewed in the context of the broader problem of the uninformed and recipe-like implementation of statistical modeling and inference, in conjunction with its many different ways it can contribute to the untrustworthiness of empirical evidence, including (a–c), and the fact that replicability is neither necessary nor sufficient for the trustworthiness of empirical evidence, the above argument is unpersuasive. The threshold $p(x_{0})\;<\;\alpha$ was never meant to be either arbitrary or fixed for all frequentist tests, and the above discussion of the large *n* problem shows that using α=0.05 for a large *n*, say n> 10,000, will often give rise to spurious significance results. Aware of the loss of power when α=0.05 decreases to α=0.005 , Benjamin et al. [6] call for increasing n, to ensure a high power of 0.8 at some arbitrary $\theta \;=\;\theta_{1}$ . The problem with the proposed remedy is twofold. First, increasing *n* is often impracticable with observational data, and second, securing high power for arbitrary discrepancies $\gamma_{1}\;=\;\left(\theta_{1}-\theta_{0}\right)$ is not conducive to learning about $\theta^{*}$ .

Another argument for lowering the threshold put forward by Benjamin et al. [6] stems from a misleading comparison between the two-sided *p*-value for the thresholds 0.05 and 0.005, and the corresponding Bayes factor:

$$\left(a\right)B_{01}\left(x_{0}\right)=\left[\pi \left(\theta \in \Theta_{0}|x_{0}\right)/\pi \left(\theta \in \Theta_{1}|x_{0}\right)\right]/\left[\pi \left(\theta \in \Theta_{0}\right)/\pi \left(\theta \in \Theta_{1}\right)\right],\forall \theta \in \Theta ,$$

where π(θ) and $\pi (\theta |x_{0})$ denote the prior and the posterior distributions. This is an impertinent comparison since the Bayesian perspective on evidence, based on $B_{01}\left(x_{0}\right),$ has a meager connection to the *p*-value as an indicator of discordance between $x_{0}$ and $\theta_{0}\;=\;0.5.$ This is because the presumed comparability (analogy) between the tail areas of $\tau \left(X\right)\overset{\mu =\mu_{0}}{∽}\mathrm{St}\left(n-1\right)$ that varies over $x\in R_{X}^{n}$ , and revolves around $\theta^{*},$ with the ratio in $B_{01}\left(x_{0}\right)$ that varies with ∀θ∈Θ, is ill-thought-out! The uncertainty accounted for by the former has nothing to do with that of the latter, since the posterior distribution accounts for the uncertainty stemming from the prior distribution, weighted by the likelihood function, both of which vary over θ .

### 4.3. The Severity Perspective on the p-Value, Observed CIs, and Effect Sizes

**The** ***p*****-value and the accept/reject** $H_{0}$ **results**. The real problem is their binary nature created by the threshold α , which gives rise to counter-intuitive results, such as for α=0.05 one rejects $H_{0}$ when $p(x_{0})\;=\;0.49,$ and accepts $H_{0}$ when $p(x_{0})\;=\;0.51.$ The SEV-based evidential account does away with this binary dimension since its inferential claim and the discrepancy γ from $\theta \;=\;\theta_{0}$ warranted with high probability are guided by the sign and magnitude of the observed test statistic $d(x_{0})$ . The SEV evaluation uses the statistical context in (15), in conjunction with the statistical approximations framed by the relevant sampling distribution of d(X) to deal with the binary nature of the results, as examples 2–4 illustrate.

**Example 2** (continued). Let us return to the data denoting newborns in Cyprus, 5152 boys and 4717 girls during 1995, where the optimal test $T_{\alpha }^{>}$ with $\;d(x_{0})\;=\;4.379,$ indicates ‘reject $H_{0}$ ’ with $p_{>}\left(x_{0}\right)\;=\;0.000006.$ When $p_{>}\left(x_{0}\right)$ is viewed from the severity vantage point, it is directly related to evaluating the post-data severity for a zero discrepancy, i.e., $\theta_{1}\;=\;\theta_{0}$ since (Mayo and Spanos, [31]):

$$\begin{array}{c} \mathrm{Sev}\left(T_{\alpha }^{>};x_{0};\theta \;>\;\theta_{0}\right)\;=\;P\left(d\left(X\right)<d\left(x_{0}\right);\theta \leq \theta_{0}\right)\;=\;1-P\left(d\left(X\right)\;>\;d\left(x_{0}\right);\theta \;=\;\theta_{0}\right)\;=\;0.99999,\; \end{array}$$

which suggests that a small *p*-value indicates the existence of *some* discrepancy γ≥0, but provides *no information* about its *magnitude* warranted by $x_{0}.$ The severity evaluation remedies that by outputting the missing magnitude in terms of the discrepancy γ warranted by data $x_{0}$ and test $T_{\alpha }^{>}$ with high probability by taking into account the relevant statistical context in (15). The key problem is that the *p*-value is evaluated using $d\left(X\right)\overset{\theta \;=\;\theta_{0}}{∽}$ N (0,1) , and thus, it contains no information relating to different discrepancies from $\theta \;=\;\theta_{0}$ , unlike the post-data severity evaluation, since it is based on ( $\sqrt{V(\theta_{1})}{)}^{-1}\left[d\left(X\right)-\delta \left(\theta_{1}\right)\right]\overset{\theta \;=\;\theta_{0}}{∽}$ N (0,1) .

**Observed CIs and effect sizes**. The question that arises is why the claim

$\mu^{*}\in CI\left(x_{0}\right)\;=\;[{\bar{x}}_{n}-c_{\frac{\alpha }{2}}\left(s\left(x_{0}\right)/\sqrt{n}\right),\;{\bar{x}}_{n}+c_{\frac{\alpha }{2}}\left(s\left(x_{0}\right)\right]$ with probability $\left(1-\alpha \right)$ is unwarranted. As argued in Section 2.4 this stems from the fact that *factual* reasoning is baseless post-data, and thus, one cannot assign a probability to $CI(x_{0})$ . This calls into question calls by the reformers in the replication crises to replace the *p*-value with the analogous observed CI because the latter is (i) less vulnerable to the large *n* problem, and (ii) more informative than the *p*-value since it provides a measure of the ‘effect size’. Cohen’s [39] recommendation is: “routinely report effect sizes in the form of confidence intervals” (p. 1002).

Claim (i) is questionable because a CI is equally as vulnerable to the large *n* problem as the *p*-value since the expected length of a consistent CI shrinks to zero as n→∞. In the case of (7) this takes the form

$E(\left[{\bar{X}}_{n}+c_{\frac{\alpha }{2}}\left(\frac{s}{\sqrt{n}}\right)\right]-\left[{\bar{X}}_{n}-c_{\frac{\alpha }{2}}\left(\frac{s}{\sqrt{n}}\right)\right])\;=\;2c_{\frac{\alpha }{2}}\left(\frac{\sigma }{\sqrt{n}}\right)\underset{n\to \infty }{\to }0,$ and thus, as *n* increases, the width of the observed $CI\left(x_{0}\right)\;=\;\left[{\bar{x}}_{n}-c_{\frac{\alpha }{2}}\left(\frac{s}{\sqrt{n}}\right),\;{\bar{x}}_{n}+c_{\frac{\alpha }{2}}\left(\frac{s}{\sqrt{n}}\right)\right]$ decreases.

This also questions claim (ii), that it provides a reliable measure of the ‘effect size’, since the larger the *n* the smaller the observed CI. Worse, the concept of the ‘effect size’ was introduced partly to address the large *n* problem using a measure that is free of *n*: “…the raw size effect as a measure is that its expected value is independent of the size of the sample used to perform the significance test.” (Abelson [40], p. 46).

**Example 2** (continued). The effect size for θ is known as Cohen’s $g\;=\;(\hat{\theta }\left(x_{0}\right)-\theta_{0})$ (Ellis [30]). When evaluated using the Arbuthnot value $\theta_{0}\;=\;0.5$ , g=0.02204 , which is rather small, and when evaluated using the Bernoulli value $\theta_{0}\;=\;\left(18/35\right)$ it is even smaller, g=0.0078 . How do these values provide a better measure of the ‘scientific effect’? They do not, since Cohen’s *g* is nothing more than another variant of the unwarranted claim that ${\hat{\theta }}_{n}\left(x_{0}\right)≃\theta^{*}$ for a large enough *n*; see Spanos [7]. On the other hand, the post-data severity evaluation outputs the discrepancy $\gamma^{‡}$ warranted by data $x_{0}$ and test $T_{\alpha }^{>}$ with probability 0.9. This implies that the severity-based effect size is $\theta_{1}^{‡}\leq 0.5156,$ which takes into account the relevant error probabilities that calibrate the uncertainty relating to the single realization $x_{0}$ . In addition, the SEV evaluation gives rise to identical severity curves (Figure 1) and the same evidence for both null values $\theta_{0}\;=\;0.5$ and $\theta_{0}\;=\;\left(18/35\right).$

**Severity and observed CIs**. The great advantage of hypothetical reasoning is that it applies both pre-data and post-data, enabling the SEV to shed light on several foundational problems relating to frequentist inference more broadly. In particular, the severity evaluation of ‘reject $H_{0}$ ’ relating to the inferential claim $\mu \;>\;\mu_{1}\;=\;\mu_{0}+\gamma_{1},\;\gamma_{1}\;\geq \;0,$ has a superficial resemblance to $CI_{L}\left(x_{0}\right)\;=\;\left(\mu \geq {\bar{x}}_{n}-c_{\alpha }\left(\frac{\sigma }{\sqrt{n}}\right)\right)$ in (8)-(b), especially if one were to consider $\mu_{1}\;=\;{\bar{x}}_{n}-c_{\alpha }\left(\frac{\sigma }{\sqrt{n}}\right)$ as a relevant discrepancy of interest in the SEV evaluation; see Mayo and Spanos [31]. This resemblance, however, is more apparent than real since:

(i)The relevant sampling distribution for $CI_{L}\left(X;\alpha \right)$ is $\tau \left(X;\mu \right)\overset{{\theta =\theta }^{*}}{∽}\mathrm{St}\left(n-1\right),$ and that for the SEV evaluation is $\tau \left(X\right)\;=\;\frac{\sqrt{n}\left({\bar{X}}_{n}-\mu_{0}\right)}{s}\overset{\mu =\mu_{1}}{∽}$ St $\left(\delta_{1},n-1\right)$ , $\forall \mu \;>\;\mu_{1}\;=\;\mu_{0}\;+\;\gamma_{1},\;\gamma_{1}\;\geq \;0$ ;

(ii) They are derived under two different forms of reasoning, factual and hypothetical, respectively, which are not interchangeable. Indeed, the presence of $\delta_{1}$ for the SEV evaluation renders the two assignments of probabilities very different. Hence, any attempt to relate the SEV evaluation to the illicit assignment $P_{L}\left(\mu \geq {\bar{x}}_{n}-c_{\alpha }\left(\frac{\sigma }{\sqrt{n}}\right)\right)\;=\;1-\alpha ,$ is ill-thought-out since (a) it will (implicitly) impose the restriction $\delta_{1}\;=\;0$ , since $\mu_{1}\;=\;{\bar{x}}_{n}-c_{\alpha }\left(\frac{\sigma }{\sqrt{n}}\right)$ leaves no room for the discrepancy $\gamma_{1},$ and (b) the assigned faux probability will be unrelated to the coverage probability; see Spanos [29]. Also, the SEV evaluation can be used to shed light on several confounds relating to different attempts to assign probabilities to different values of θ within an observed CI, including the most recent attempt based on confidence distributions by Schweder and Hjort [20] above.

## 5. The SEV Evaluation vs. the Law of Likelihood

Royall [11] popularized an alternative approach to converting inference results into evidence using the likelihood ratio anchored on the Maximum Likelihood (ML) estimator ${\hat{\theta }}_{n}\left(x_{0}\right)$ . He rephrased Hacking’s [41] *Law of Likelihood (LL)*: *Data* $x_{0}$ *support hypothesis* $H_{0}$ *over hypothesis* $H_{1}$ *if and only if* $L\left(H_{0};x_{0}\right)>L\left(H_{1};x_{0}\right).$ *The degree to which* $x_{0}$ *supports* $H_{0}$ *better than* $H_{1}$ *is given by the Likelihood Ratio (LR):*

$$\begin{array}{c} LR\left(H_{0},H_{1};x_{0}\right)\;=\;L\left(H_{0};x_{0}\right)/L\left(H_{1};x_{0}\right),\; \end{array}$$

by replacing the second sentence with “The likelihood ratio $LR(H_{0},H_{1};x_{0})$ measures the strength of evidence for $H_{0}$ ”. The term “strength of evidence" is borrowed from Fisher’s [42] questionable claim about the *p*-value: “The actual value of *p* … indicates the *strength of evidence* against the hypothesis” (p. 80).

To avoid the various contradictions arising from allowing $H_{0}$ or $H_{1}$ to be composite hypotheses (Spanos, [32,43]), Royall’s account of evidence revolves around simple hypotheses $\theta_{0}$ vs. $\theta_{1}.$ This, however, creates a problem with nuisance parameters that does not arise in the context of N-P testing where all the parameters θ of the invoked $M_{\theta }\left(x\right)$ are viewed as an integral part of the inductive premises, irrespective of whether any one parameter is of particular interest.

To exemplify the Royall LR (RLR) account of evidence let us return to example 2.

**Example 2** (continued). Consider the data 5152 boys $\left(X\;=\;1\right)$ and 4717 girls $\left(X\;=\;0\right)$ in the context of the simple Bernoulli model in (18), yielding the Maximum Likelihood (ML) estimate ${\hat{\theta }}_{n}\left(x_{0}\right)\;=\;0.52204$ of θ . For $y_{n}\;=\;\sum_{i=1}^{n}x_{i}$ the likelihood function takes the form:

$$\begin{array}{c} L\left(\theta ;x_{0}\right)\propto f\left(x_{0};\theta \right)\;=\;\theta^{y_{n}}{\left(1-\theta \right)}^{n-y_{n}}\;=\;\theta^{5152}{\left(1-\theta \right)}^{4717},\;\theta \in \left(0,1\right) \end{array}$$

**Figure 7:**
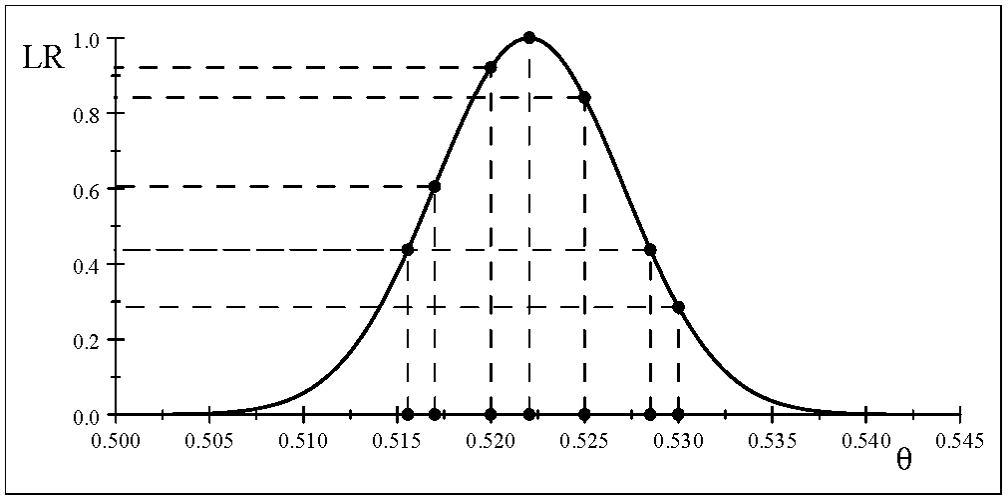
The RLR strength of evidence for $\theta_{0}\;=\;$ 0.52204 vs. $\theta_{1}\in \left(0.5,0.545\right)$ .

The likelihood function scaled by the ML estimate is (Figure 7):

$$\begin{array}{c} L_{s}\left(\theta ;x_{0}\right)\;=\;\frac{{\left(\theta \right)}^{5152}{\left(1-\theta \right)}^{4717}}{{\left(0.52204\right)}^{5152}{\left(1-0.52204\right)}^{4717}},\;\theta \in \left(0,1\right)\; \end{array}$$

Consider the hypotheses $H_{0}$ : $\theta_{0}\;=\;0.52204$ vs. $H_{1}$ : $\theta_{1}\;=\;0.51557$ whose likelihood ratio yields:

$$\begin{array}{c} LR\left(\theta_{0},\theta_{1};x_{0}\right)\;=\;\frac{{\left(0.52204\right)}^{5152}{\left(1-0.52204\right)}^{4717}}{{\left(0.51557\right)}^{5152}{\left(1-0.51557\right)}^{4717}}\;=\;2.286. \end{array}$$

The first issue that arises is how to interpret 2.286 in terms of the strength of evidence. Royall [11] proposes three thresholds:

$$\begin{array}{cc} \mathrm{weak}: & LR(\theta_{0},\theta_{1};x_{0})\leq 4,\\ \mathrm{fairly}\;\mathrm{strong}: & 4<LR(\theta_{0},\theta_{1};x_{0})\leq 8,\\ \mathrm{very}\;\mathrm{strong}: & LR(\theta_{0},\theta_{1};x_{0})\geq 32. \end{array}$$

Irrespective of how justified these thresholds are in the proposer’s mind, they can be easily disputed as ad hoc and arbitrary since the likelihood function $L\left(\theta ;x_{0}\right)\propto f\left(x_{0};\theta \right)$ , as well as the LR $LR(\theta_{0},\theta_{1};x_{0}),$ depend crucially on the invoked statistical model $M_{\theta }\left(x\right).$ Hence, when $LR(\theta_{0},\theta_{1};x_{0})$ is used to determine the appropriate distance between the two likelihoods, the notion of universal thresholds is dubious. This is because every likelihood function has an in-built natural distance relating to each of its parameters known as *the score function*: the derivative of the log-likelihood $s\left(\theta \right)\;=\;\frac{d\ln L(\theta ;x)}{d\theta },\;x\in R_{X}^{n}$ , evaluated at a particular point $\theta \;=\;\theta_{1}$ . Its key role stems from the fact that its mean is zero and its variance is equal to Fisher’s information; see Casella and Berger [19]. The score function indicates the sensitivity of lnL(θ;x) to infinitesimal changes of $\theta_{1}$ . The problem is that the score function differs for different statistical models. For instance, for the simple Normal in (2) the score function $s\left(\mu \right)\;=\;\frac{n({\bar{X}}_{n}-\mu )}{\sigma^{2}}$ is linear in μ , but for the simple Bernoulli model in (18) the score function $s\left(\theta \right)\;=\;\frac{n({\bar{X}}_{n}-\theta )}{\theta (1-\theta )}$ is highly non-linear in θ , even though both parameters denote the mean of their underlying distributions. This calls into question the notion of LR universal thresholds, and could explain why so many different such thresholds have been proposed in the literature; see Reid [44].

To shed light on Royall’s strength of evidence notion, Table 5 reports $LR(\theta_{0},\theta_{1};x_{0})$ for $\theta_{0}\;=\;0.52204$ and different values of $\theta_{1},$ including the value related to the SEV warranted discrepancy, $\gamma_{1}^{‡}\;=\;0.01557$ [ $\theta_{1}\;=\;0.51557$ ] with probability 0.9 , as shown in Figure 7.

Using the above thresholds, the result $LR(\theta_{0},\theta_{1};x_{0})\;=\;2.269$ indicates that the strength of evidence for $\theta_{0}\;=\;0.52204$ vs. $\theta_{1}\;=\;0.51557$ is ‘weak’, and the evidence will be equal or weaker for any $\theta_{1}\in \left(0.51557,0.5285\right)$ . If one were to take a firm stand on $x_{0}$ providing ‘fairly strong’ evidence for $\theta_{0},$ the relevant range of values for $\theta_{1}$ will be $\theta_{1}\notin \left(0.5117,0.5323\right)$ .

What is even more problematic for the RLR approach is that $\theta_{0}\;=\;0.52204$ has the same strength of evidence against the two values $\theta_{1}\;=\;0.51557$ and $\theta_{2}\;=\;0.5285$ , shown in Figure 7. This calls into question the nature of evidence the RLR approach gives rise to since it undermines the primary objective of frequentist inference. Its strength of evidence for $\theta_{0}$ is identical against two values on either side of the ML estimate value 0.52204, and as the threshold increases the distance between them increases. This derails any learning from data since it undermines the primary objective of narrowing down the relevant neighborhood for $\theta^{*}$ unless the choice is invariably the ML point estimate as it relates to the fallacious claim ${\hat{\theta }}_{n}\left(x_{0}\right)≃\theta^{*}$ . This weakness was initially pointed out by an early pioneer of the likelihood ratio approach, Barnard [45], p. 129, in his belated review of Hacking [41]. He argued that for any prespecified value $\theta_{1}$ :

“… there always is such a rival hypothesis, viz. that things just had to turn out the way they actually did.”

As evidenced in Figure 7, the RLR will pinpoint the ML estimate $\hat{\theta }\left(x_{0}\right)$ no matter what the other value in $\left(0,1\right)$ happens to be since it is *the maximally likely value*; see Mayo [46]. No wonder, Hacking [47], p. 137, in his book review of Edward’s [48] “ Likelihood” changed his mind and abandoned the LR approach altogether:

“I do not know how Edwards’s favoured concept [the difference of log-likelihoods] will fare. The only great thinker who tried it out was Fisher, and he was ambivalent. Allan Birnbaum and myself are very favourably reported in this book for things we have said about likelihood, but Birnbaum has given it up and I have become pretty dubious.”

Indeed, Hacking [49], p. 141, not only rejected the Law of Likelihood but went a step further by reversing his original viewpoint and wholeheartedly endorsing the N-P testing:

“This paper will show that the Neyman-Pearson theories of testing hypotheses and of confidence interval estimation are sound theories of probable inference.”

It is worth noting that even before the optimal N-P theory of testing was finalized in 1933, Pearson and Neyman [50] confronted the problem of how to construe $LR(\theta_{0},\theta_{1};x)$ by emphasizing the crucial role of the relevant error probabilities:

“But if we accept the criterion suggested by the method of the likelihood it is still necessary to determine its sampling distribution in order to control the errors involved in rejecting a true hypothesis, a knowledge of λ [ $LR(\theta_{0},\theta_{1};x_{0})$ ] alone is not adequate to insure control of the error. … In order to fix a limit between "small" and "large" value of λ we must know how often such values appear when we deal with a true hypothesis.” (p. 106).

A critical weakness of RLR approach is that learning from data about $\theta^{*}$ using two points $LR(\theta_{0},\theta_{1};x_{0})$ at a time becomes untenable since the parameter space is usually uncountable, and thus the point ${\hat{\theta }}_{n}\left(x_{0}\right)\;=\;\theta^{*}$ will always belong to a set of measure zero; see Williams [51]. In addition, the ‘maximally likely value’ problem is compounded by the fact that the framing of $LR(\theta_{0},\theta_{1};x_{0})$ runs afoul of the two crucial stipulations [1]–[2] introduced by Neyman and Pearson [23] in Section 2.3.

A likely counter-argument might be that the RLR approach ignores any potentially relevant error probabilities, but asymptotically the likelihood function will pinpoint $\theta^{*}$ , by invoking the Strong Law of Large Numbers (SLLN):

(29) $$\;\begin{array}{c} P\left(\lim_{n\to \infty }\left[\ln(\frac{\frac{1}{n}L_{n}\left(\theta^{*};x\right)}{\frac{1}{n}L_{n}\left(\theta ;x\right)})\right]>0\right)\;=\;1,\;\forall \theta \in \Theta ,\;L_{n}\left(\theta ;x\right)\;=\;\sum_{i=1}^{n}\ln f\left(x;\theta \right),\;\forall x\in R_{X}^{n}.\; \end{array}$$

This argument is misplaced since it does not address the key problems relating to $LR(\theta_{0},\theta_{1};x_{0})$ narrowing down the potential neighborhood of $\theta^{*}$ , to give rise to any learning from data. To begin with, as the above quote from Le Cam [27], such limits theorems are uninformative as to what happens with the particular data. Also, the probabilistic assignment in (29) relies on $\forall x\in R_{X}^{n},$ revolves around $\theta^{*}$ , and has nothing to do with $x_{0}$ and $L_{n}\left(\theta_{i};x_{0}\right),\;i\;=\;0,1.$ Indeed, for the same reason the Kullback-Leibler (K-L) divergence (Lele, [52] in [53]) defines the difference between $L_{n}\left(\theta_{0};x\right)$ and $L_{n}\left(\theta_{1};x\right),$ $\forall x\in R_{X}^{n}$ , and provides a comparative account relative to $f(x_{0};\theta )$ , and not $f\left(x;\theta^{*}\right),\;x\in R_{X}^{n},$ as specified by (29). This implies that the RLR and the K-L divergence invoke (implicitly) a variant of the fallacious claim ${\hat{\theta }}_{n}\left(x_{0}\right)≃\theta^{*}$ .

A closer look at the RLR approach to evidence reveals that $LR(\theta_{0},\theta_{1};x_{0})$ provides nothing more than a ranking of all the different pairs of values of θ in (0,1) relative to ${\hat{\theta }}_{n}\left(x_{0}\right)$ , regardless of the ‘true’ value $\theta^{*}.$ This can be easily demonstrated using simulation with a known $\theta^{*}$ to show that the RLR search using different replications $x_{i},\;i\;=\;1,2,\ldots ,N$ , and the associated ML estimates ${\hat{\theta }}_{n}\left(x_{i}\right),\;i\;=\;1,2,\ldots ,N$ , is unlikely that any one of them will be equal or very close to the true value. To get anything close to $\theta^{*}$ one should use all the replications for a sufficiently large *N* to approximate closely the sampling distribution, $f\left({\hat{\theta }}_{n}\left(x\right);\theta \right),\;x\in R_{X}^{n},$ of ${\hat{\theta }}_{n}\left(X\right)$ , and use the overall average $\frac{1}{N}\sum_{i=1}^{N}{\hat{\theta }}_{n}\left(x_{k}\right)≃\theta^{*}.$ This, however, is based on *N* data sets of sample size *n* not just the one, $x_{0}$ . Hence, the ratio $LR(\theta_{0},\theta_{1};x_{0})$ contains no information relating to $f\left({\hat{\theta }}_{n}\left(x\right);\theta \right),\;x\in R_{X}^{n}$ , beyond being a single observation. In this sense, the RLR is as unduly data-dependent as the point estimate ${\hat{\theta }}_{n}\left(x_{0}\right)$ in (16) and the associated observed CI in (17) since it also ignores the uncertainty stemming from ${\hat{\theta }}_{n}\left(x_{0}\right)$ being a single observation from $f\left({\hat{\theta }}_{n}\left(x\right);\theta \right),\;x\in R_{X}^{n}.$

More importantly, comparing the results of the SEV evaluation in Table 1 with those based on $LR(\theta_{0},\theta_{1};x_{0})$ in Table 5 indicates clearly that the two approaches to evidence are incompatible. The SEV evaluation relating to the ML estimate ${\hat{\theta }}_{n}\left(x_{0}\right)$ will always yield 0.5 , rendering it unwarranted in principle! Worse, the SEV evaluation of the discrepancy associated with $\theta_{1}\;=\;0.51557$ is warranted with probability 0.9 , but the value $\theta_{2}\;=\;0.5285$ , that the RLR approach assigns the same ‘strength of evidence’ relative to $\theta_{0}\;=\;0.52204$ is warranted with probability 0.099! Which of the two approaches gives rise to pertinent evidence stemming from constituting a sound inductive generalization of the relevant statistical results, the accept/reject for the SEV, and the ML estimate for the RLR?

One can make a credible case that the ML estimate ${\hat{\theta }}_{n}\left(x_{0}\right)$ , would lie in some broad neighborhood of $\theta^{*}$ , but narrowing that down sufficiently to learn about $\theta^{*}$ cannot be attained using $LR(\theta_{0},\theta_{1};x_{0})$ . This stems from the fact that the RLR approach revolves around the ML estimate which is based on an *estimation perspective* that cannot be deployed post-data since $X=x_{0}$ has occurred, and thus $\theta \;=\;\theta^{*}$ has transpired. In contrast, the SEV evaluation is based on a *testing perspective*, which is equally pertinent for evaluating error probabilities pre-data and post-data.

The most crucial feature of the SEV evaluation is that it converts the accept/reject $H_{0}$ results into evidence by taking into account the statistical context in (15) including the power of the test. This is important since detecting a particular discrepancy, say $\gamma_{1}$ , provides stronger evidence for its presence when the power is lower than higher; see Spanos [18]. The underlying intuition can be illustrated by imagining two people searching for metallic objects on the same beach, one is using a highly sensitive metal detector that can detect small nails, and the other a much less sensitive one. If both detectors begin to buzz simultaneously, the one more likely to have found something substantial is the less sensitive one! The SEV evaluation harnesses this intuition by custom-tailoring the power of the test, replacing the original $c_{\alpha }$ with the post-data $d(x_{0})$ , to establish the discrepancy $\gamma_{1}$ warranted by data $x_{0}$ with high enough probability. As argued above, this enables the SEV evidential account to circumvent several foundational problems, including the large *n* problem. In contrast, the RLR account will yield the same strength of evidence for any two values of θ , irrespective of the size of *n*.

## 6. Summary and Conclusions

The replication crisis has exposed the apparent untrustworthiness of published empirical evidence, but its narrow attribution to certain abuses of frequentist testing can be called into question as ‘missing the forest for the trees’. A stronger case can be made that the real culprit is the much broader problem of the *uninformed and recipe-like, implementation* of statistical methods, which contributes to the untrustworthiness in many different ways, including [a] imposing invalid probabilistic assumptions on one’s data, and [b] conflating unduly data-specific inference results’ with ‘evidence for or against inferential claims about $\theta^{*}$ ’, which represent inductive generalizations of such results.

The above discussion makes a case that the post-data severity (SEV) evaluation provides an evidential account of the accept/reject $H_{0}$ results, in the form of a discrepancy γ≠0 from $\theta \;=\;\theta_{0},$ warranted with high enough probability by data $x_{0}$ and test $T_{\alpha }$ . The SEV evaluation is framed in terms of a post-data error probability that accounts for the statistical context in (15), as well as the uncertainty stemming from inference results relying on a single realization of the sample $X\;=\;x_{0}$ . The SEV evaluation perspective is used to call into question Royall’s [11] LR approach to evidence as another rendering of the fallacious claim ${\hat{\theta }}_{n}\left(x_{0}\right)≃\theta^{*}$ for a large enough n.

The SEV evaluation is also shown to elucidate/address several foundational issues confounding frequentist testing since the 1930s, including (i) statistical vs. substantive significance, (ii) the large *n* problem, and (iii) the alleged arbitrariness of the N-P framing $H_{0}$ and $H_{1}$ , used to undermine the coherence of frequentist testing. The SEV also oppugns the proposed alternatives to replace or modify frequentist testing, using statistical results, such as observed CIs, effects sizes, and redefining significance, all of which are equally vulnerable to [a]–[c] undermining the trustworthiness of empirical evidence. In conclusion, it is important to reiterate that unless one has already established the statistical adequacy of the invoked $M_{\theta }\left(x\right)$ , any discussions relating to reliable inference results and trustworthy evidence based on tail area probabilities are unwarranted.

## Figures and Tables

**Table 1:** SEV for ‘reject $\theta_{0}\;=\;0.5$ ’ and ‘accept $\theta_{0}\;=\;\left(18/35\right)$ ’ with ( $T_{\alpha }^{>};x_{0})$ .

$\theta_{0}\;=\;0.5$ : $\gamma_{1}\;=\;$	0.01	0.014	0.015	0.01557	0.016	0.018	0.022	0.025	0.03
$\theta_{0}\;=\;\frac{18}{35}$ : $\gamma_{1}\;=\;$	−0.0043	−0.0003	0.0007	0.00128	0.0017	0.0037	0.0077	0.0107	0.016
$\theta_{1}\;=\;$	0.51	0.514	0.515	0.51557	0.516	0.518	0.522	0.525	0.53
$\mathrm{Sev}(\theta \;>\;\theta_{1})=$	0.991	0.944	0.918	0.900	0.884	0.787	0.500	0.276	0.056

**Table 2:** Standardized α(n) relative to $\left(\alpha \;=\;0.05,n\;=\;100\right)$ .

*n*	50	100	200	500	1000	5000	10,000	${1\;\times \;10}^{5}$	${1\;\times \;10}^{6}$	${1\;\times \;10}^{7}$	${1\;\times \;10}^{8}$
α(n)	0.071	0.05	0.035	0.022	0.016	0.007	0.005	0.0016	0.0005	0.00016	0.00005

**Table 3:** The *p*-value with increasing *n* ( ${\bar{x}}_{n}$ constant).

*n*	2000	3000	3256	5000	10,000	20,000	50,000	100,000
$d_{n}\left(x_{0}\right)$	1.971	2.414	2.515	3.117	3.652	6.234	9.856	13.939
$p_{n}\left(x_{0}\right)$	0.0341	0.0128	0.0099	0.0019	0.000023	$4.1\;\times \;10^{-9}$	$3.9\;\times \;10^{-20}$	0.000000⋯

**Table 4:** SEV with changing *n* ( ${\bar{x}}_{n}\;=\;0.52204$ is held constant).

*n*	3256	5000	10,000	12,000	20,000	50,000	100,000
$d_{n}\left(x_{n}\right)$	2.515	3.117	4.441	4.828	6.234	9.856	13.938
$SEV(\theta \;>\;\theta_{1};n)\;=\;0.9$ : $\theta \;>\;\theta_{1}$	0.5109	0.513	0.5156	0.5162	0.5175	0.5192	0.5200
SEV(θ>0.51557;n)=	0.770	0.820	0.903	0.922	0.967	0.998	0.999

**Table 5:** RLR strength of evidence for $\theta_{0}\;=\;0.52204$ vs. different values of $\theta_{1}$ .

$\theta_{1}=$	0.5085	0.51	0.513	0.514	0.51557	0.525	0.5285	0.532	0.534	0.536
$LR(\theta_{0},\theta_{1})\;=\;$	37.36	17.5	5.02	3.59	2.286	1.189	3.51	6.57	17.02	47.59
$[1/LR\left(\theta_{0},\theta_{1}\right)]\;=\;$	0.027	0.057	0.199	0.276	0.437	0.841	0.437	0.152	0.059	0.021

## Data Availability

All data used are available in published sources.

## Abbreviations

The following abbreviations are used in this manuscript:

N-P	Neyman–Pearson
MDS	Medical diagnostic screening
M-S	Misspecification
CI	Confidence interval
SEV	Post-data severity
